# Putative degraders of low‐density polyethylene‐derived compounds are ubiquitous members of plastic‐associated bacterial communities in the marine environment

**DOI:** 10.1111/1462-2920.15232

**Published:** 2020-09-28

**Authors:** Maria Pinto, Paula Polania Zenner, Teresa M. Langer, Jesse Harrison, Meinhard Simon, Marta M. Varela, Gerhard J. Herndl

**Affiliations:** ^1^ Department of Functional and Evolutionary Ecology University of Vienna Vienna Austria; ^2^ Research Platform ‘Plastics in the Environment and Society’, University of Vienna Vienna Austria; ^3^ Centre for Microbiology and Environmental Systems Science University of Vienna Vienna Austria; ^4^ Institute for Chemistry and Biology of the Marine Environment (ICBM), University of Oldenburg Oldenburg Germany; ^5^ IEO, Instituto Español de Oceanografía, Centro Oceanográfico de A Coruña A Coruña Spain; ^6^ NIOZ, Department of Marine Microbiology and Biogeochemistry Royal Netherlands Institute for Sea Research, Utrecht University Den Burg The Netherlands

## Abstract

It remains unknown whether and to what extent marine prokaryotic communities are capable of degrading plastic in the ocean. To address this knowledge gap, we combined enrichment experiments employing low‐density polyethylene (LDPE) as the sole carbon source with a comparison of bacterial communities on plastic debris in the Pacific, the North Atlantic and the northern Adriatic Sea. A total of 35 operational taxonomic units (OTUs) were enriched in the LDPE‐laboratory incubations after 1 year, of which 20 were present with relative abundances > 0.5% in at least one plastic sample collected from the environment. From these, OTUs classified as *Cognatiyoonia*, *Psychrobacter*, *Roseovarius* and *Roseobacter* were found in the communities of plastics collected at all oceanic sites. Additionally, OTUs classified as *Roseobacter*, *Pseudophaeobacter*, *Phaeobacter*, *Marinovum* and *Cognatiyoonia*, also enriched in the LDPE‐laboratory incubations, were enriched on LDPE communities compared to the ones associated to glass and polypropylene in in‐situ incubations in the northern Adriatic Sea after 1 month of incubation. Some of these enriched OTUs were also related to known alkane and hydrocarbon degraders. Collectively, these results demonstrate that there are prokaryotes capable of surviving with LDPE as the sole carbon source living on plastics in relatively high abundances in different water masses of the global ocean.

## Introduction

Between 4.8 and 12.7 million metric tons of plastic are entering the ocean each year (Jambeck *et al*., 2015). Like all solid surfaces in the ocean, plastics are colonized by microbial communities referred to as the plastisphere (Zettler *et al*., 2013), which is different in its composition from the microbial communities found in the surrounding environment (Lobelle and Cunliffe, 2011). This has been shown for plastics collected in different locations (Amaral‐Zettler *et al*., 2015; Oberbeckmann *et al*., 2015; De Tender *et al*., 2017; Kettner *et al*., 2017; C Dussud *et al*., 2018b; Harrison *et al*., 2018), and has been observed in both seawater (SW) (Amaral‐Zettler *et al*., 2015) and sediment habitats (Harrison *et al*., 2014).

The composition of the plastisphere is also shaped by biogeographic and environmental factors, such as salinity and nutrient availability (Oberbeckmann *et al*., 2014; 2018; Amaral‐Zettler *et al*., 2015; Kesy *et al*., 2019). Surface characteristics of the plastic, such as surface free energy, hydrophobicity, presence of additives, weathering state and molecular composition might also play a role in determining the community composition of plastic‐associated biofilms (Gross *et al*., 2016; Cai *et al*., 2019; Hossain *et al*., 2019), especially in earlier stages of biofilm development (Pinto *et al*., 2019). Because some marine bacteria have the ability to degrade hydrocarbons, it has been suggested that a certain fraction of the microbial community colonizing plastics could also be capable of degrading plastics (Dussud *et al*., 2018a; Jacquin *et al*., 2019; Roager and Sonnenschein, 2019). For example, the bacterium *Ideonella sakaiensis* produces enzymes capable of efficiently converting poly(ethylene‐terephthalate) (PET) into benign monomers (Yoshida *et al*., 2016).

The two most abundant plastics in the ocean, polyethylene (PE) and polypropylene (PP), show signs of degradation when incubated with specific bacteria or fungi under controlled laboratory conditions (Sudhakar *et al*., 2008; Harshvardhan and Jha, 2013; Paço *et al*., 2017). Whether these organisms also degrade marine plastics in the oceanic environment, however, remains unclear. The majority of the tested microorganisms, such as *Bacillus* sp. (Harshvardhan and Jha, 2013) and the fungus *Zalerion maritimum* (Paço *et al*., 2017), while isolated from SW, were not particularly abundant in plastic‐associated biofilms, indicating that they likely play only a minor role in plastic degradation in the marine environment.

However, weight loss has been reported for PE and PP during in situ incubations in the marine environment, indicating either an abiotic loss due to fragmentation and leaching or biotically via microbial action on plastics (Sudhakar *et al*., 2007). The systematic association of bacteria capable of degrading hydrocarbons, such as members of the families *Alcanivoraceae*, *Rhodobacteraceae* and *Sphingomonadaceae* (Oberbeckmann and Labrenz, 2019; Roager and Sonnenschein, 2019) to plastic surfaces further suggests that specific members of plastic‐colonizing microbial communities associated with this plastic might be capable of degrading plastic and/or plastic‐derived compounds in the ocean. For example, the family *Alcanivoraceae*, well known for including alkane‐degrading taxa (Schneiker *et al*., 2006; Kim *et al*., 2018), has been found enriched on PET surfaces compared to glass surfaces (Oberbeckmann *et al*., 2016). *Alcanivoraceae* also increase in relative abundance in incubations where plastics, such as low‐density polyethylene (LDPE) and polystyrene, are the only carbon sources (Delacuvellerie *et al*., 2019; Syranidou *et al*., 2019). Weight loss of plastics and changes of the chemical properties of their surface, when incubated with specific microorganisms, are indicative of plastic biodegradation (Oberbeckmann and Labrenz, 2019). However, except for *I. sakaiensis* producing PET‐degrading enzymes (Yoshida *et al*., 2016), the metabolic pathways and associated mechanistic processes involved in the biodegradation of other plastics are yet to be characterized. Therefore, whether oceanic bacteria are capable to degrade plastics remains to be shown.

Recently, the taxonomy of plastic‐associated microbial communities has been studied in the Mediterranean Sea (Dussud *et al*., 2018), the Baltic Sea (Oberbeckmann *et al*., 2014; 2018; Kettner *et al*., 2017; Ogonowski *et al*., 2018; Kesy *et al*., 2019), the North Sea (Oberbeckmann *et al*., 2014; 2016; De Tender *et al*., 2017; Kirstein *et al*., 2019), the northern Adriatic Sea (Pinto *et al*., 2019), the Caribbean Sea (Dudek *et al*., 2020), the western Atlantic Ocean (Zettler *et al*., 2013; Amaral‐Zettler *et al*., 2015; Debroas *et al*., 2017) and the North Pacific Gyre (Amaral‐Zettler *et al*., 2015; Bryant *et al*., 2016). The composition of plastic‐associated bacterial communities has been compared using materials collected in the North Sea, the Baltic Sea and Yangtze Estuary (Oberbeckmann and Labrenz, 2019). The community composition of the samples from each of these locations was obtained by the taxonomic classification of the V4 region of the 16S rRNA gene (De Tender *et al*., 2017; Jiang *et al*., 2018; Ogonowski *et al*., 2018; Oberbeckmann *et al*., 2018; Kesy *et al*., 2019). While the biofilm samples from different locations did share operational taxonomic units (OTUs), mostly affiliated to *Rhodobacteraceae* and *Sphingomonadaceae* families, the majority of OTUs were location‐specific. This suggests that environmental variables are the main factor determining the composition of prokaryotic plastic‐associated communities within marine habitats. Most of these studies, however, used different DNA extraction methods, different primers and different sequencing technologies, which might introduce biases in the results and might explain some of the differences in community composition found between locations (Wintzingerode *et al*., 1997).

In this study, we compare the composition of prokaryotic communities associated with plastics collected in the Pacific, the North Atlantic and the northern Adriatic Sea (Fig. S1; Table S1). If biodegradability of plastics is a factor influencing the composition of plastic‐colonizing prokaryotic communities in the ocean, we expect the presence of plastic degraders in plastic‐associated biofilms to be independent of the oceanic region. Furthermore, it has been proposed that metabolic pathways involved in the biodegradation of plastics differ between different plastic polymers (Devi *et al*., 2016). Hence, we hypothesized that different plastic types (e.g. PE, PP) would harbour different putative plastic‐degrading organisms. To determine whether specific bacteria originating from the natural microbial community can utilize LDPE‐derived compounds, we incubated two PE biofilm communities with LDPE as their sole carbon source, one for 1 year and the other for 2 years. We then investigated the presence of the bacteria increasing in relative abundance in these incubations and on plastics collected in different regions of the global ocean. We also examined whether the bacteria enriched in the LDPE incubations were present in biofilms associated with PE, PP and glass during an in‐situ incubation experiment taking place in the northern Adriatic Sea (described in Pinto *et al*. 2019). In this previous study, we characterized the composition of bacterial communities on glass and different plastic types after 1 week, 1 month and 2 months using the same molecular and sequencing tools and protocols as used in this study.

## Results

### Community composition across different oceanic regions

We analysed the 16S rRNA gene sequences of biofilms associated with 13, 23 and 40 polymer (plastic) items collected in the North Atlantic, Pacific and northern Adriatic Sea respectively (Table S2). We could not identify the polymer type of 1 and 6 pieces from the North Atlantic and Pacific respectively. Thus, they were classified as not identified (NI).

Overall, OTU diversity and richness were significantly higher on plastics collected in the northern Adriatic than in the North Atlantic and Pacific, independent of the size of the plastics (Fig. S2; Tables S3 and S4).

**Fig 1:**
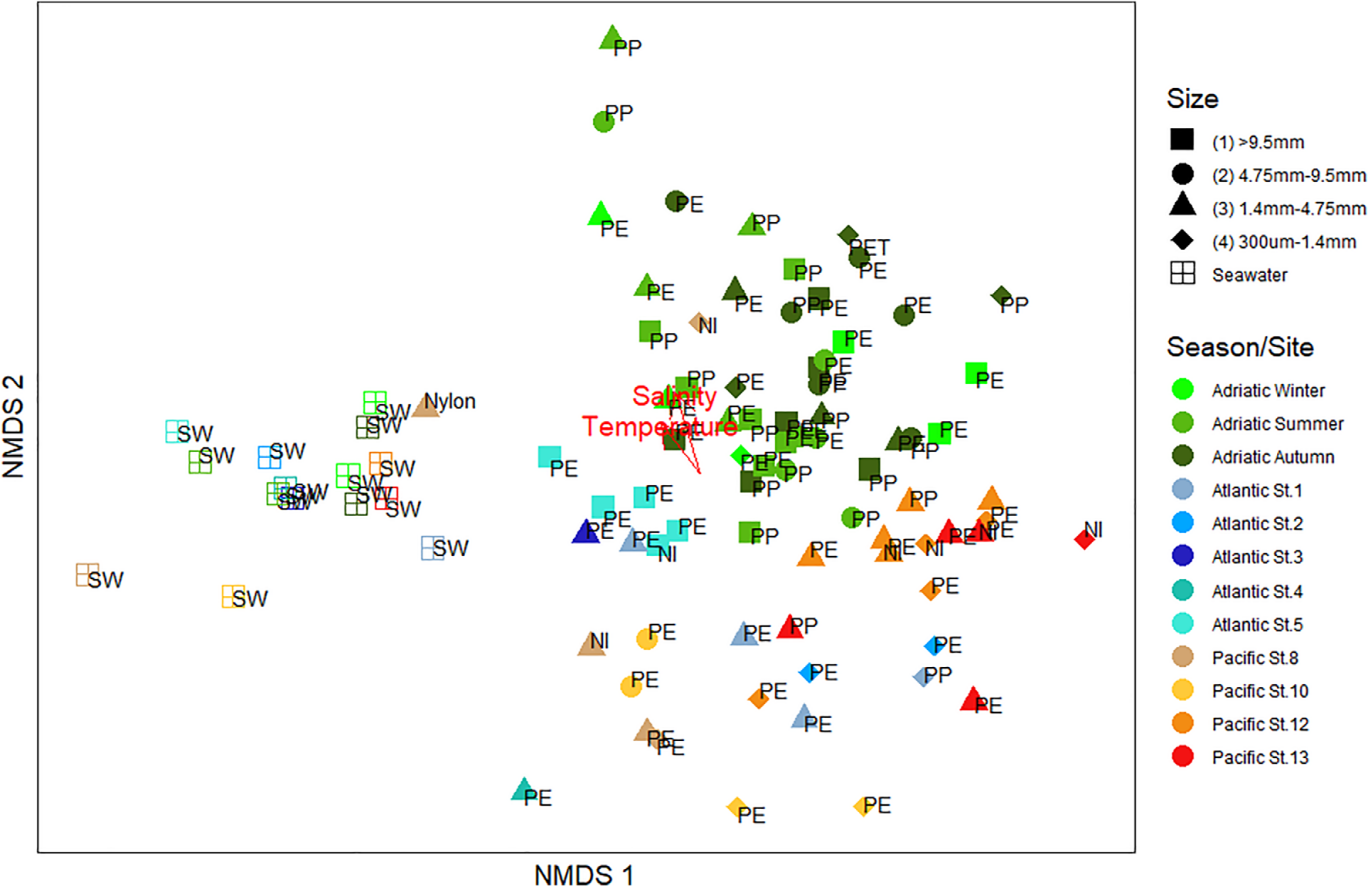
Non‐metric multidimensional scaling (NMDS) representation of the prokaryotic community of all plastic and seawater samples collected in the northern Adriatic, North Atlantic and Pacific using Bray‐Curtis as the distance measurement. Each symbol represents one sample and the letters next to each symbol indicate the type of plastic (PE, PP, PET and nylon), unidentified polymers (NI) or seawater (SW). The environmental factor vectors were fitted to the NMDS through the envfit function. Envfit results are given in Table S6.

There was no difference between the taxonomic composition of the bacterial communities colonizing PP and PE (PERMANOVA: *F* = 0.97, *p* = 0.527). The composition of bacterial communities on plastics, however, significantly differed from that of the surrounding SW at all sites (Fig. 1; Table S5). Furthermore, the bacterial community composition in plastic‐associated biofilms differed between the oceanic basins, i.e. North Atlantic, Pacific and northern Adriatic, and between sampling stations, albeit not in all of them (Fig. 1; Table S5). The bacterial communities on plastics collected in the Pacific at Stations 8 and 10 were relatively similar but were significantly different from the communities on plastics collected at any other Pacific stations (Table S5). The bacterial community composition of the plastic‐associated biofilms collected in the North Atlantic was relatively similar, with the exception of plastics collected at Stations 1 and 5, which significantly differed from each other (PERMANOVA: *F* = 1.50, *p* < 0.05).

When we fitted environmental vectors to the non‐metric multidimensional scaling (NMDS) scores of all samples, we obtained significant correlations for temperature and salinity (*p* < 0.05), but only when ambient SW communities were included (Fig. 1; Table S6). When considering only the northern Adriatic and North Atlantic biofilm samples, NMDS scores were significantly correlated with temperature and NO_2_, NO_3_ and PO_4_ concentrations (Table S6). However, when examining the correlation between environmental factors and the overall (combined) community composition of biofilm samples from each station, Mantel tests indicated that there was no significant correlation with any of the environmental factors (Table S7). The bacterial community composition of the plastic‐associated biofilm collected in the North Atlantic was significantly correlated with the distance between stations (*R* ^2^ = 0.697, *p* < 0.05). For more details on the taxonomy of the bacterial community on plastics and SW from the different sampling sites, see Supporting Information S1.

We identified 462 OTUs shared between plastics across the different oceanic regions, 57 of them were present only on plastics (Fig. S6A; for taxonomy, see Table S9). When considering only PE biofilms, there were 427 shared OTUs. A total of 471 OTUs with > 1% relative abundance was present in at least one sample at each site, distributed between plastics and SW from the Atlantic, Pacific and northern Adriatic (Fig. S6B; for taxonomy; see Table S10). Out of these 481 OTUs, only 22 OTUs exhibited a relative abundance of > 1% in the plastic biofilm at all three oceanic basins (Fig. S6B; for taxonomy, see Table S11) and only 10 OTUs were also shared with ambient SW. Together, these 19 OTUs contributed a varying percentage to the total classified 16S rRNA gene sequences on plastics collected at the different locations, with a minimum of 7% in one plastic sample collected at Station 4 in the North Atlantic and a maximum of 51% ± 0.04% (*n* = 3) on plastics collected at Station 13 in the Pacific.

Bacterial communities associated with plastics collected in the northern Adriatic exhibited a higher number of unique OTUs with a relative abundance > 1% (260 OTUs) than corresponding samples collected in the North Atlantic and Pacific, where only 13 and 41 unique OTUs were found respectively (Fig. S6).

#### Taxa enriched on plastic‐associated communities across different oceanic regions

**Fig 2:**
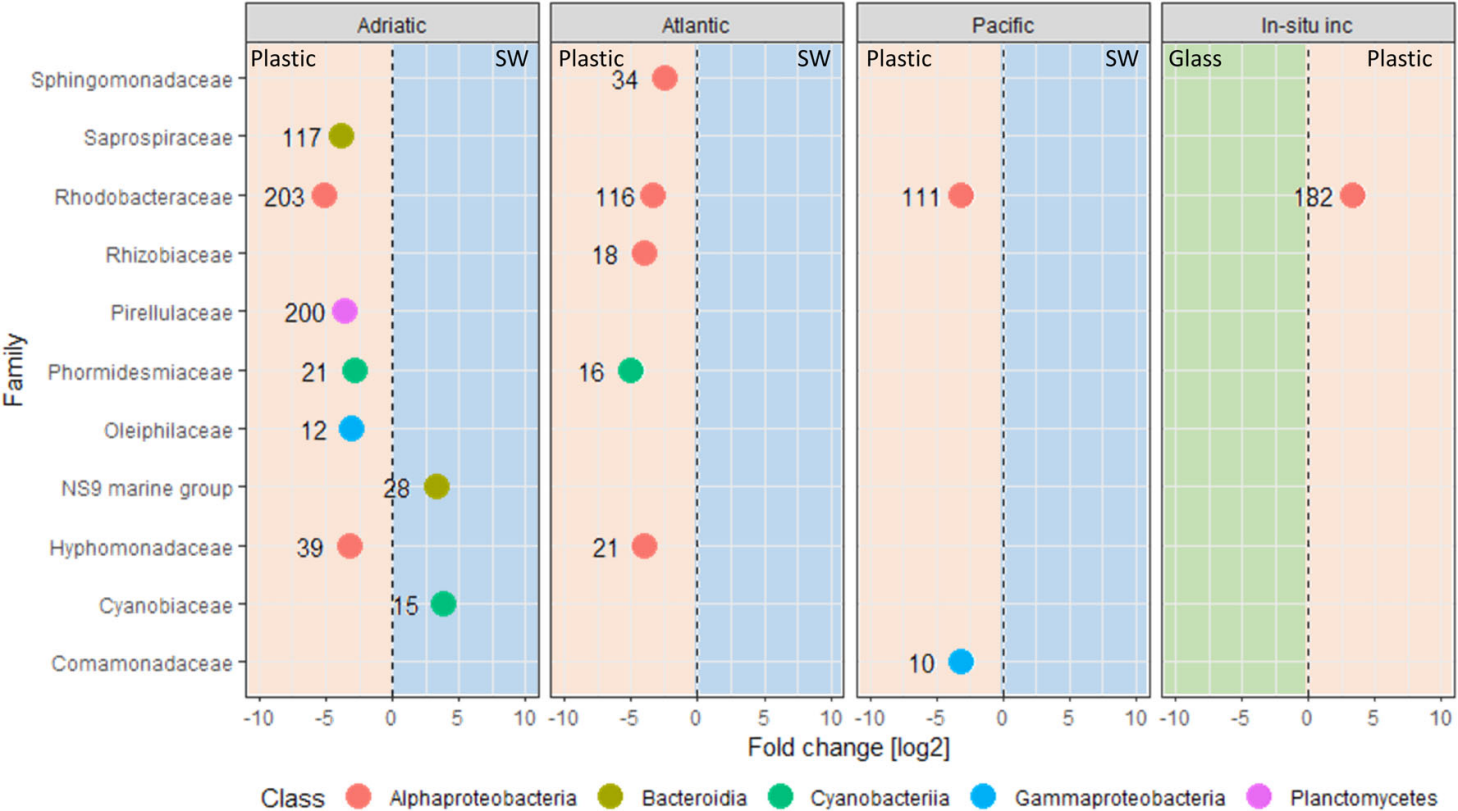
Enriched bacterial families between plastics and seawater in the northern Adriatic, the North Atlantic and the Pacific, and between plastics (HDPE + LDPE + PP) and glass in the in‐situ incubations (in situ inc.) in the northern Adriatic. Only significantly enriched taxa were included (adjusted *p* < 0.05). Families enriched on plastics, glass and in seawater (SW) are represented in the red, green and blue areas respectively. The number associated with each symbol represents the number of OTUs in the family.

We identified taxa enriched on plastics in comparison to SW in the different water masses using DESeq2 analysis. Communities associated to plastics collected in the northern Adriatic harboured more significantly enriched families compared to SW than plastics collected in the North Atlantic and Pacific. The family *Rhodobacteraceae* was the only family enriched on plastics in all three oceanic regions (Fig. 2). The families *Phormidesmiaceae* and *Hyphomonadaceae* were also enriched on plastics collected in the northern Adriatic and North Atlantic compared to the corresponding SW. The families *Saprospiraceae*, *Pirellulaceae* and *Oleiphilaceae* were enriched only on plastics collected in the northern Adriatic compared to the ambient water, while *Sphingomonadaceae* and *Rhizobiaceae* were enriched only on plastics collected in the North Atlantic, and *Comamonadaceae* were enriched only on plastics collected in the Pacific compared to the corresponding ambient water (Fig. 2).

The bacterial community associated with PE and PP in the northern Adriatic was enriched in some OTUs (Fig. S7). Four *Flavobacteriaceae* OTUs (OTU 2231, OTU 243, OTU 300 and OTU 5577), one *Microtrichaceae* OTU (OTU 1048), one *Oleiphilaceae* OTU (OTU 49), one *Rhodobacteraceae* OTU (OTU 1457) and three *Sphingomonadaceae* OTUs (OTU 36, OTU 393 and OTU 6729) were enriched on PE, and one *Phormidesmiaceae* OTU (OTU 38), one *Flavobacteriaceae* OTU (OUT 137) and one *Saccharospirillaceae* OTU (OUT 61) were enriched on PP.

#### Taxa enriched on PE, PP or glass in in‐situ incubations in the northern Adriatic

To identify taxa enriched on PE versus PP versus glass, we also performed the DESeq2 analysis on 16S rRNA amplicon sequences from the 2 month incubation experiment of Pinto *et al*. (2019). *Rhodobacteraceae* was the only enriched family on plastic (PE and PP) compared to glass in the in‐situ incubations.

**Fig 3:**
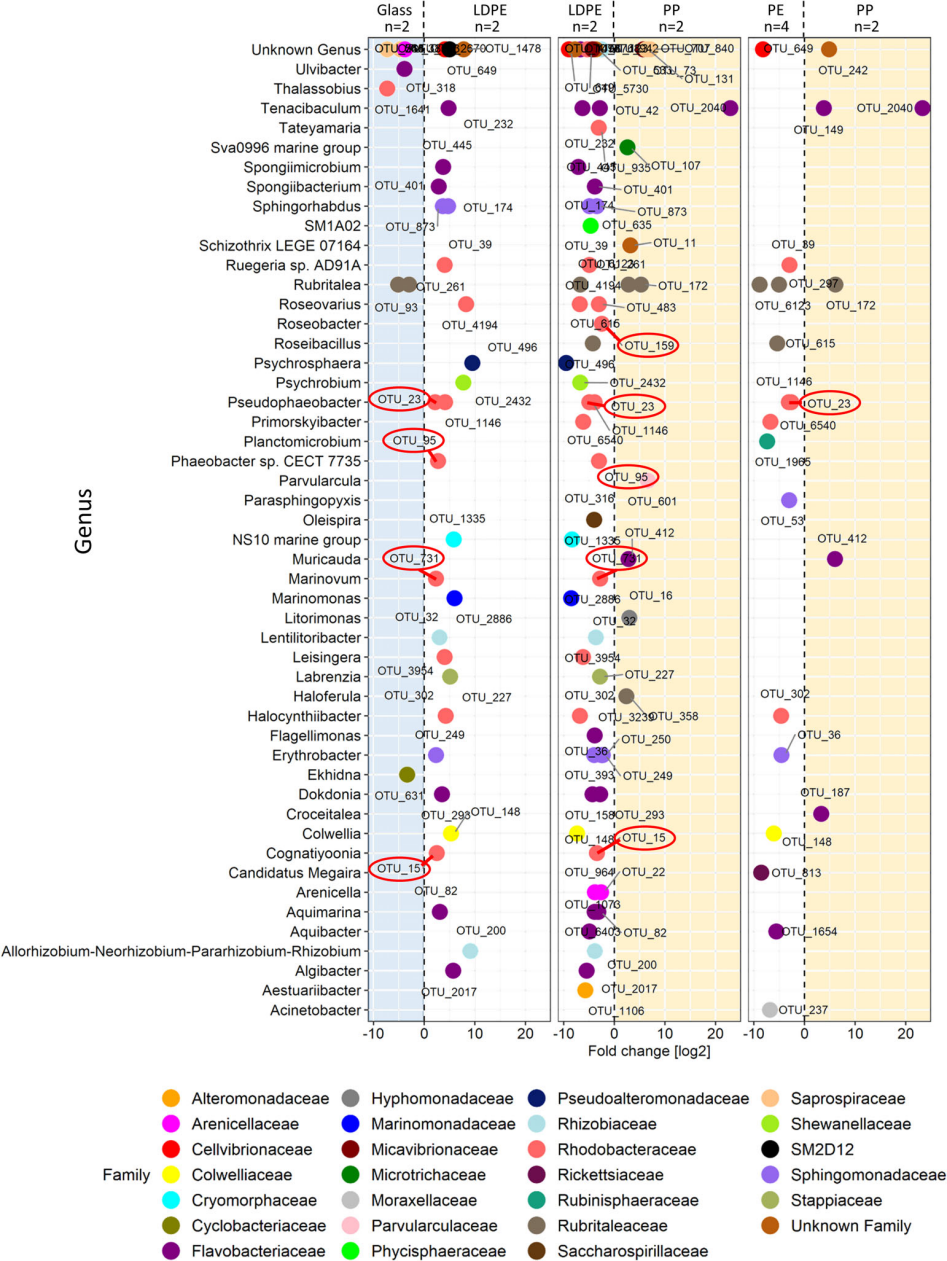
Enriched bacterial families between different surfaces incubated in the northern Adriatic after a 1 month incubation (Pinto *et al*., 2019). Only significantly enriched taxa are included (adjusted *p* < 0.05). Each symbol represents one OTU. OTUs enriched on glass are represented in the blue area, OTUs enriched on PE (LDPE + HDPE) and LDPE are represented in the white area and OTUs enriched in PP are represented in the orange area. The red circles indicate OTUs also enriched in abundance in the LDPE laboratory incubations after 1 year.

Comparing the bacterial communities on PE (LDPE+HDPE) and glass of the three sampling dates combined, there were no OTUs enriched on either one of the surfaces (DESeq2 analysis, *p* > 0.05). When performing the same analysis comparing the bacterial communities of PE and PP, OTU 6540, classified as *Primorskyibacter sedentarius*, was enriched in PE (log_2_ Fold Change = −5.53, *p* < 0.05). However, when analysing only the samples incubated for 1 month in the in‐situ incubations, there were 10 OTUs enriched on the PE (LDPE + HDPE) over glass, and two OTUs enriched on glass over PE. Interestingly, there were four *Rhodobacteraceae* OTUs (OTU 731, OTU 15, OTU 95 and OTU 23) enriched in LDPE biofilms over both PP and glass biofilms (Fig. 3).

#### LDPE‐laboratory incubations

We incubated two plastics (Fig. S8) collected in the northern Adriatic (incubations A and B) in artificial SW amended with ammonium and phosphate for 2 years with LDPE as the sole carbon source. A total of 35 OTUs (Table S12) increased in relative abundance in these incubations after 1 year compared to the composition of the initial inoculum. These OTUs were also still present in incubation B after 2 years. Out of these 35 OTUs, only 19 were present in the incubations after one or 2 years with relative abundances > 0.5% (Fig. S9). Operational taxonomic units (OTUs) classified as *Cognatiyoonia* (OTU 15), *Parvibaculaceae* (OTU 28), *Cognatishimia* (OTU 40), *Methyloligellaceae* (OTU 55), *Ketobacter* (OTU 60) and *Sneathiella* (OTU 273) were present in both incubations A and B in relative abundances > 1% after 1 year and also after 2 years in incubation B (Fig. S8).

**Fig 4:**
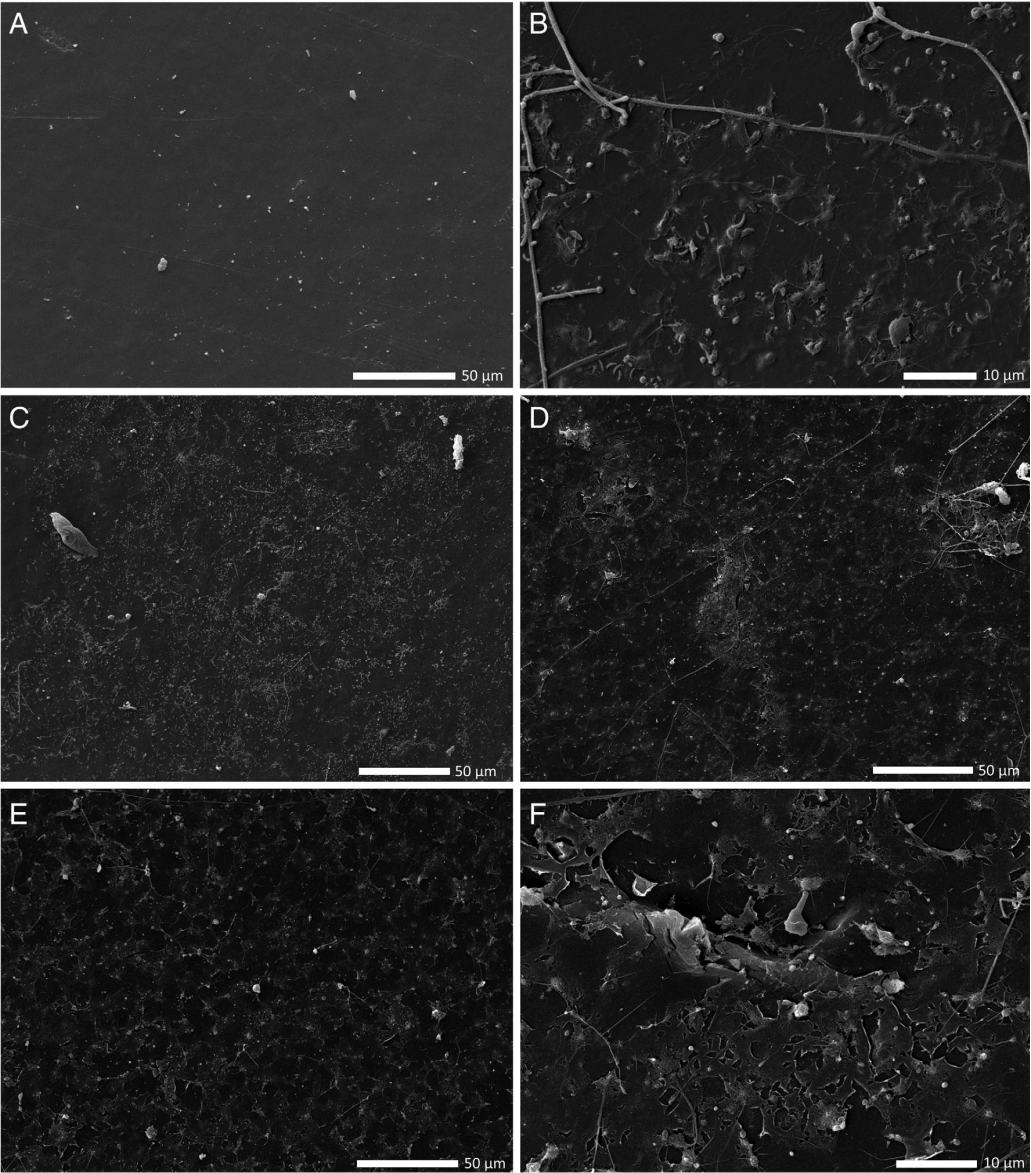
Scanning electron microscopy (SEM) pictures of (A) a control plastic incubated in artificial seawater without the addition of a plastic previously covered with a biofilm; (B) plastic A 1 year after the initial incubation; (C) plastic B 1 year after the initial incubation; (D) plastic A 2 years after the initial incubation; (E) plastic B 2 years after the initial incubation and (F) plastic A 1 year after the initial incubation after biofilm removal.

Scanning electron microscopy (SEM) analysis revealed the presence of a biofilm on both incubations A and B after one and 2 years (Fig. 4B–E). After removing the biofilm from a plastic piece from incubation A after 1 year, holes on the plastics surface were visible (Fig. 4F).

### Presence of the OTUs enriched in the LDPE‐laboratory incubations on plastics in the ocean

We searched for the 35 OTUs enriched in our LDPE‐laboratory incubations on the plastics and SW collected in the three oceanic regions and on the in‐situ incubation in the northern Adriatic Sea. These OTUs represented 4.1 ± 3.2%, 4.8 ± 4.8% and 7.4 ± 6.4% of the total bacterial community associated with plastics collected in the northern Adriatic, the North Atlantic and the Pacific respectively. Sixteen OTUs were present on at least one plastic at all three sampling sites (Fig. S6C).

**Fig 5:**
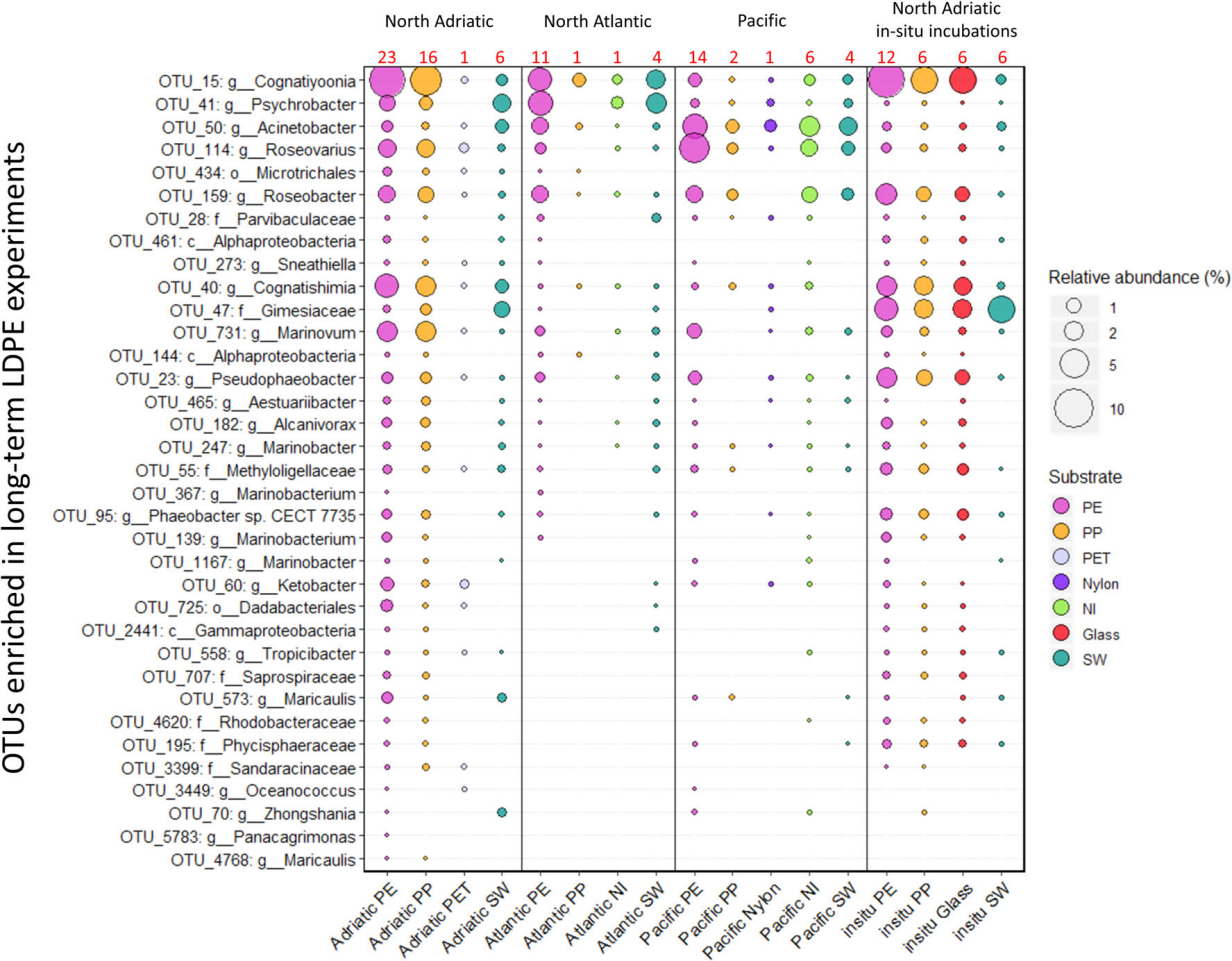
Relative abundance of OTUs enriched in the LDPE laboratory long‐term incubations in seawater and different surfaces across the different sampling sites. The numbers in red indicate the number of samples. The OTUs represented on the y‐axis are the ones enriched on LDPE biofilms after 1 year of incubation in artificial seawater with LDPE as their sole carbon source.

**Fig 6:**
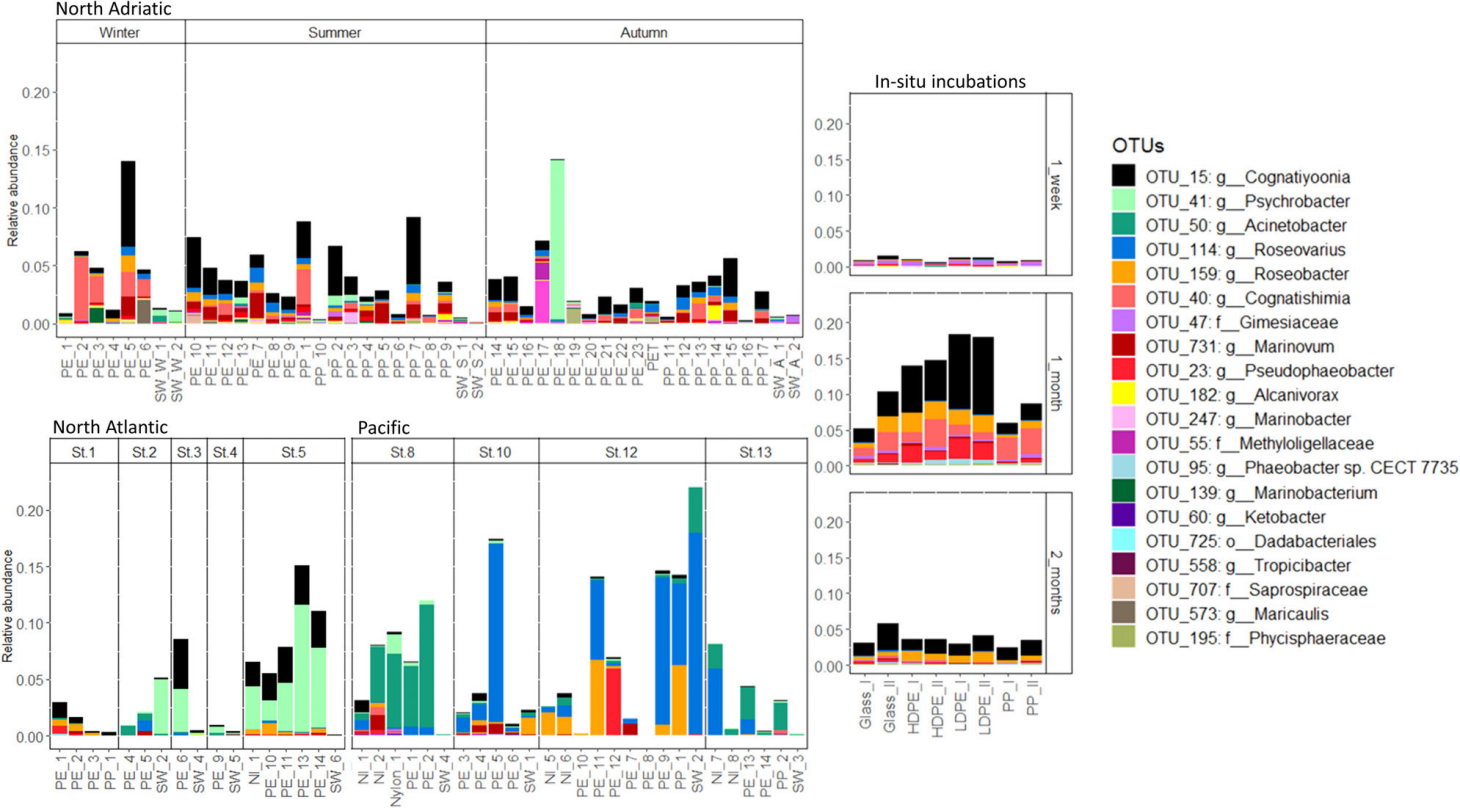
Stacked plots of the relative abundance of the OTUs enriched in the long‐term LDPE incubations after 1 year incubated in ASW with LDPE as sole carbon source. Only OTUs that occurred with > 0.5% relative abundance in at least one of the samples are shown. The x‐axis represents each individual sample collected in the northern Adriatic, North Atlantic, Pacific and on the different surfaces incubated for 2 months in the northern Adriatic (Pinto *et al.*, 2019). The y‐axis indicates the relative abundance of the classified 16S rRNA genes from 0% to 23%. The labels represent the number of the OTU followed by its lowest taxonomic classification.

From the 35 OTUs, representatives of *Cognatiyoonia*, *Psychrobacter*, *Acinetobacter*, *Roseovarius*, *Roseobacter* and *Marinovum* were found to be relatively abundant on plastics at all three locations (Fig. 5). OTU 15, affiliated to *Cognatiyoonia*, was found on almost all plastic at all stations (Fig. 6), however, with varying relative abundances of up to 7%. In the ambient SW, the highest relative abundance of this representative (OTU 15) of the *Cognatiyoonia* was only 0.8%.

This difference, however, was not detectable anymore after 2 months in the in‐situ incubation. From the 35 OTUs, representatives of *Cognatiyoonia* and *Psychrobacter* (OTUs 15 and 41 respectively) were the two most abundant OTUs in the plastic associated biofilm in the North Atlantic (Fig. 5). *Acinetobacter* and *Roseovarius* (OTUs 50 and 114 respectively) were the two most abundant OTUs in the biofilm from plastics collected in the Pacific (Fig. 5).

The four *Rhodobacteraceae* OTUs enriched on LDPE sheets in the in‐situ incubations (OTU 731, OTU 15, OTU 95 and OTU 23) when compared to PP and glass after 1 month of incubation were also enriched in the laboratory LDPE‐incubations (Fig. 6). However, this enrichment was not detectable anymore after 2 months of incubation. OTU 15 was particularly abundant on LDPE after 1 month of incubation compared to the biofilm of PP and glass. OTU 159, also enriched in the laboratory incubations, was also enriched on LDPE compared to PP. OTU 36, classified as *Erythrobacter*, was enriched on PE over PP in both the Adriatic and in the in‐situ incubations after 1 month of incubation (DESeq2 plot PE versus PP Adriatic).

## Discussion

There has been discussion on whether there is a core plastic biofilm community and whether plastic degraders are part of it (Debroas *et al*., 2017). Our study reveals that while there is high variability in bacterial community composition even between plastics collected at the same site, certain taxa are consistently present on plastics even when collected in different oceanic regions (Fig. 1). Some of these taxa that are ubiquitous on plastics were enriched in our incubations with LDPE as the sole carbon source, suggesting that they might utilize LDPE‐derived compounds as a carbon source.

The bacterial community composition on PE and PP was different from that of the ambient water (Fig. 1), similar to what has been shown in previous studies (Dussud *et al*., 2018; Amaral‐Zettler *et al*., 2020) but was relatively similar between the two polymers at the different sites. For a more detailed discussion on the comparison of the bacterial community composition of plastic‐associated biofilms collected at the different sites, see Supporting Information S2. Some OTUs, however, were preferentially colonizing or growing on either PE or PP in the northern Adriatic (Fig. S6). For example, OTUs classified as *Erythrobacter* sp. (OTU 36, OTU 6729 and OTU 393) and *Oleiphilus* sp. (OTU 49) were enriched in PE over PP. Members of these genera have been shown to harbour genes encoding alkane monooxygenases and to play an important role in oil degradation in the ocean (Wang *et al*., 2010; Toshchakov *et al*., 2017). Alkane degradation pathways have been suggested as the most probable PE degradation pathway given the similar chemical structure of PE and alkanes (Devi *et al*., 2016). Interestingly, OTU 36 (affiliated to *Erythrobacter* sp.) was also enriched on PE compared to PP in the in situ incubations after 1 month (Fig. 4). Furthermore, *Erythrobacter* sp. has been previously found to be more abundant on PE than on any other surfaces. In the Baltic Sea, *Erythrobacter* sp. was also abundant especially on PE (Oberbeckmann *et al*., 2018), and one *Erythrobacter* OTU was exclusively associated with PE collected in the Baltic and the North Sea (Oberbeckmann and Labrenz, 2019). The enrichment of alkane‐degraders on PE in comparison to PP is consistent with the possibility that certain organisms discriminate for certain plastics either due to their ability to degrade plastic‐derived hydrocarbons or utilize specific plastic additives.

Members of the *Sphingomonadaceae*, such as *Erythrobacter* sp., and *Rhodobacteraceae* have been regularly found associated with plastics (Bryant *et al*., 2016; Debroas *et al*., 2017; Curren and Leong, 2019). Additionally, Oberbeckmann and Labrenz (2019) found that some OTUs from these families made up more than half of the only 45 OTUs present on plastics collected in four different studies. Our study provides further evidence that members of the *Rhodobacteraceae* family are in fact enriched on plastics compared to SW and other surfaces across different water masses. This is not surprising since members of the *Rhodobacteraceae* family are key members of biofilms developing on different surfaces in the marine environment (Elifantz *et al*., 2013). As revealed in the in‐situ incubations, however, some of them, such as *Cognatiyoonia* sp. (OTU 15), *Pseudophaeobacter* sp. (OTU 23), *Phaeobacter* sp. (OTU 95) and *Roseobacter* sp. (OTU 159) have a preference for LDPE over glass and PP. This might be due to their ability to degrade LDPE‐related hydrocarbons as indicated by their enrichment in our laboratory incubations with LDPE as the sole carbon source after 1 year. However, whether they have the ability to degrade the LDPE hydrocarbon backbone, associated plastic additives (Wright *et al*., 2020) or passively released compounds (Romera‐Castillo *et al*., 2018) remains unknown. Furthermore, it is important to note that only duplicate samples were collected in the in situ incubations in the northern Adriatic, precluding rigorous statistical analyses.

*Cognatiyoonia* is a new genus within the *Rhodobacteraceae* family (Wirth and Whitman, 2018) to reclassify *Loktanella koreensis* and *Loktanella sediminum*. The genus *Loktanella* has been previously found to be enriched on plastics over non‐plastic surfaces (Ogonowski *et al*., 2018) and particularly abundant on PET and LDPE (Delacuvellerie *et al*., 2019). In our study, OTU 15, classified as *Cognatiyoonia*, was present in the biofilm of almost all plastics (Fig. 6). Also, OTU 15 exhibited a high relative abundance in the laboratory incubations with LDPE as the sole organic carbon source after 1 year. This might indicate that OTU 15 has the ability to utilize plastic‐derived hydrocarbons, especially those related to alkanes. Certain *Loktanella* strains have the ability to degrade n‐alkanes between C_10_ and C_22_ (Harwati *et al*., 2007). Furthermore, they have been found enriched in oil degradation experiments (Lanfranconi *et al*., 2010; Sanni *et al*., 2015). The potential of *Cognatiyoonia* sp. to degrade hydrocarbons and plastics is uncertain, but its ubiquitous presence on plastics collected at different locations and the ability to survive on LDPE as sole carbon source suggest that it might play a role in the degradation of plastics in the ocean.

The above‐mentioned genera *Pseudophaeobacter* sp. (OTU 23), *Phaeobacter* sp. (OTU 95) and *Roseobacter* sp. (OTU 159), also enriched in the long‐term incubations with LDPE as the only carbon source, have also been associated with hydrocarbon degradation (Bacosa *et al*., 2018; Brakstad *et al*., 2018). Even though they were present in lower relative abundance than OTU 15, they were still present on plastics collected at all the different oceanic regions (Fig. 6).

As suggested by the in‐situ incubation, the relative abundance of the OTUs enriched in the LDPE‐laboratory incubations varies between different stages of biofilm development, especially on LDPE. The putative LDPE‐degrading OTUs were most prevalent during the early stages of biofilm development after about 1 month of incubation and significantly decreased in relative abundance after 2 months (Fig. 6). Also, after 1 month of incubation the largest differences in bacterial community composition between different plastic types were observed in the in situ incubations (Pinto *et al*., 2019). Microbes with a preference for a specific type of plastic are dominant in the biofilm layers which are in close contact with the plastic surface (Kirstein *et al*., 2019). In subsequently formed layers when the biofilm thickens, the bacterial community is most likely shaped by internal biofilm processes and micro‐environmental factors rather than by microbes selected due to surface properties. For example, Pinto *et al*. (2019) reported that after a 1 and 2 months incubation period the composition of the bacterial communities growing on different plastics were more similar to each other than after 1 week of incubation. This might explain the decrease in relative abundance of organisms potentially capable of degrading LDPE‐originating compounds in the later stages of biofilm development. This agrees with studies reporting similar bacterial community compositions on different polymers after prolonged incubation periods (Kirstein *et al*., 2018). The decrease in the number of OTUs with preference for a specific surface in later stages of the biofilm development might be the result of changes in the biofilm environment, no longer favouring microbes with the ability to metabolize plastic‐originating compounds. The development of a thicker biofilm creates a diverse habitat with a plethora of organic carbon compounds (Dang and Lovell, 2016) probably more readily accessible to bacteria than plastic‐derived hydrocarbons (Min *et al*., 2020). Nevertheless, in the in‐situ incubations even after 2 months, OTUs enriched in our LDPE‐laboratory incubations were still present in the biofilms obtained from all plastics (Fig. 6).

Taken together, degraders of plastic‐derived compounds are recruited early in the development of the biofilm on plastic surfaces (Kirstein *et al*., 2019) and attain high relative abundance at the early stage of biofilm formation, but decrease in relative abundance with further biofilm development. These putative plastic‐compound degraders remain in the plastic‐associated biofilm in low relative abundances, potentially in the layers that are tightly connected to the plastic surface. Whether these bacteria do degrade the plastics remains unknown. For example, members of the *Rhodobacteraceae* family are known to exhibit high metabolic versatility (Michael *et al*., 2016) and are capable of occupying different metabolic niches when environmental conditions change.

In conclusion, through a combination of in situ incubations, laboratory long‐term incubations with LDPE as the sole organic carbon source and analyses of collected plastics from three oceanic basins, strong evidence is provided for the ubiquitous presence of bacteria capable of utilizing LDPE‐derived compounds as a carbon source within plastic biofilms in the global ocean.

## Experimental procedures

### Sampling

Sampling was conducted at four stations in the Pacific, five stations in the North Atlantic and at one location in the northern Adriatic Sea (Fig. S1; Table S1). The Pacific samples were collected aboard R/V *Sonne* during the SO248 expedition with a bongo net and a mesh size of 300 μm in May–June 2016. The net was kept at the sea surface by attaching one float on each side of the net (Fig. S8). The North Atlantic samples were collected aboard the R/V *Rámon Margalef* during the RadProf 2017 expedition belonging to the time series RADIALES‐PROFUNDOS in July 2017 (Prieto *et al*., 2015). The northern Adriatic Sea samples were collected in February, August and November of 2016. Samples from the North Atlantic and the northern Adriatic Sea were collected with a net for microplastic sampling (Hydrobios, Germany) with a mesh size of 500 μm. At all locations, the net was towed with 2 knots for 30–45 min (detailed information of sampling at each station is given in Table S1). Water samples (2–5 l) from all locations were also collected and immediately filtered through 0.2 μm polycarbonate filters and stored at −80°C.

The plastics were collected from the cod end of the net into a bucket filled with SW from the same location and immediately taken to the laboratory for processing. To separate the collected plastic material by size, the samples were passed through a series of sieves with mesh size of 9.5 mm, 4.5 mm, 1.4 mm and 300 μm and rinsed with 0.2 μm freshly filtered SW. The content collected in each sieve was immediately transferred to petri dishes with 0.2 μm filtered SW. Each plastic piece was collected from the Petri dishes, washed three times in 0.2 μm filtered SW to remove all organisms not tightly attached to the plastic biofilm, put in a Greiner tube and immediately stored at −80°C.

### Inorganic nutrient analyses and environmental data

Inorganic nutrient concentrations (NO_3_^−^, NO_2_^−^ and PO_4_^3−^) were determined only on SW samples collected with Niskin bottles just below the sea surface. In the northern Adriatic Sea, surface SW was filtered through combusted Whatman GF/F filters and stored in PE bottles at −20°C. Nutrient analyses were performed within 1 month following standard protocols (Strickland and Parsons, 1972). Surface SW samples collected in the North Atlantic were frozen on board until the inorganic nutrients were measured in the laboratory using a QuAAtri auto‐analyser (SEAL Analytical) following published protocols (Coverly *et al*., 2012). Most nutrient concentrations at the Pacific stations were below the detection limit using standard methods. Therefore, they were excluded from this study.

Temperature and salinity measurements from the northern Adriatic Sea were made available from the sampling station RV001 located off the coast of Rovinj by the Ruđer Bošković Centre for Marine Research at Rovinj, Croatia. Temperature and salinity data from the stations in the Pacific and North Atlantic were obtained from the sensors mounted on the CTD sampling rosette deployed at each station. Data from the Pacific are available at Pangaea (https://doi.pangaea.de/10.1594/PANGAEA.864673).

### Colonization pattern of LDPE and other plastics incubated in situ in the northern Adriatic Sea

To investigate the presence of putative LDPE degraders in biofilms on different surfaces over a 2 month incubation period in the northern Adriatic Sea, we used 16S rRNA amplicon sequencing of the prokaryotic communities collected during incubations exposed to solar radiation, downloaded from the NCBI Sequence Read Archive (SRA) database (BioProject: PRJNA515271). Details of the experimental setup are given elsewhere (Pinto *et al*., 2019).

The samples comprised duplicates of 4 cm^2^ sized pieces of virgin PP, LDPE and HDPE, with approximately 0.5 mm thickness, glass slides of 1 cm^2^, with approximately 1 mm thickness and SW samples. The different substrates were mounted on a floating frame in the northern Adriatic Sea, approximately 500 m off the coast of Rovinj, Croatia, over a 3 month period (November 2016–January 2017) and the samples were taken after 1 week, 1 month and 2 months of incubation. Processing of the samples, the DNA extraction method, PCR primers and sequencing technology and specifications used were the same as described previously (Pinto *et al*., 2019).

### Enrichment of bacterial communities living on LDPE as sole carbon source

Plastic material was collected in August 2016 off the coast of Rovinj, Croatia, and rinsed with 0.2 μm filtered SW. Two large plastic pieces (Fig. S8) were incubated each in a 1 l borosilicate bottle filled with artificial SW (ASW) amended with 10 μM NH_4_Cl and 2 μM Na_2_PO_4_ (incubations A and B), however, without an organic carbon source other than that contained in the plastic material. The incubations were held at room temperature in the dark. For a scheme of the experimental setup, see Fig. S9. After 3 weeks, each plastic was transferred to a new bottle with the same conditions. After repeating this procedure for 3 months, a small piece of the plastic of approximately 2.25 cm^2^ was cut and transferred to a new bottle with ASW amended with ammonium and phosphate added in the form of NH_4_Cl and Na_2_PO_4_, respectively, using the same concentrations as mentioned above, and 1 cm^2^ sterile LDPE sheets were added as a carbon source. The LDPE sheets were purchased from Goodfellow. The same transfer procedure was repeated with new LDPE sheets every 3 weeks for a total period of 2 years. After 1 and 2 years of the initial incubation, one square from each flask which had the biofilm developing for 1 month was taken for 16S rRNA gene amplicon sequencing of the biofilm community. Due to low quality sequencing output, the sample after 2 years of incubation A was excluded from further analysis. Two plastic pieces from the same flasks were also taken for SEM analysis after one and 2 years. One piece was used to analyse the intact biofilm, and the second was used to analyse the surface of the plastic after removal of the biofilm. To remove the biofilm, the piece was submerged in Milli‐Q water, vortexed for 30 s, then submerged in 4% SDS buffer at room temperature for 4 h. Subsequently, the plastic was submerged again two times in fresh Milli‐Q water, vortexed for 30 s and gently dried on clean paper. A control plastic that was incubated in a separate bottle with the same ASW as incubations A and B for 1 month was also stored for SEM analysis.

#### SEM analysis

Samples for SEM were incubated in 2% glutaraldehyde for 10 min and then stored at −80°C until processing. The samples were then dehydrated with an ethanol dilution series of 30%, 50%, 70%, 80%, 90%, 95% each for 10 min and three times in 100% absolute ethanol for 10 min. The dehydrated samples were CO_2_ critical‐point dried with a CPD 300 auto critical‐point dryer (Leica Microsystems). The dried pieces were gold‐coated using a JFC‐2300HR sputter coater (JEOL) for 80 s. Pictures were taken using a secondary electron detector with a JEOL JSM‐IT300 scanning electron microscope with a 15 kV acceleration voltage in ultra‐high vacuum.

### DNA extraction and 16S rRNA sequencing

DNA extraction of all samples was performed using the modified bead‐beating approach in combination with the Puregene Tissue DNA extraction kit (Qiagen, Valencia, CA) (Debeljak *et al*., 2017). DNA extraction and sequencing were performed in four batches. The first one comprised all the northern Adriatic samples, the second the laboratory LDPE incubations after 1 year incubation and the Pacific samples, and the third the laboratory LDPE incubations after 2 years incubation and the North Atlantic samples. For each batch, we added a blank that was extracted, PCR amplified and sequenced at the same time as the samples using the same protocols (Fig. S12).

The primer pair 341F (5′‐CCT ACG GGN GGC WGC AG‐3′) and 802R (5′‐TAC HVG GGT ATC TAA TCC‐3′) was used to PCR amplify the V3‐V4 hypervariable region of the 16S rRNA gene, resulting in fragments of ~460 bp. Each PCR was performed in a total volume of 52 μl containing 25 μl Library Amplification Ready Mix 2xMM (Kapa Biosystems), 1 μl of each 25 μM primer, 23 μl of sterile water and 2 μl of extracted DNA. Amplification was done on a Mastercycler Pro (Eppendorf AG, Germany). The conditions consisted of an initial denaturation at 95°C for 3 min, followed by 20 cycles at 98°C for 20 s, at 56°C for 30 s and at 72°C for 30 s, and a final elongation at 72°C for 5 min.

PCR products were then purified in a 96‐well plate using the Agencourt AMPure XP Purification kit and protocol (Beckman Coulter Life Sciences). These purified PCR products were barcoded and an additional 10‐cycle PCR was done, followed by paired‐end next‐generation amplicon sequencing (2 × 250 bp) using Illumina MiSeq technology at Microsynth AG, Switzerland. All raw sequence files were submitted to the NCBI Sequence Read Archive (SRA) database (BioProject: PRJNA623496).

### Identification of plastic types using Raman microspectroscopy

After DNA extraction, each plastic was dried on a clean paper and its chemical nature was identified using a LabRAM HR800 confocal Raman spectroscopy system (Horiba Jobin‐Yvon). Excitation for Raman scattering was provided by a 532 nm NdYAG laser. The Raman spectrum was obtained for the range of 400–3200 cm^−1^. Each sample was compared with reference scans from plastics of known composition. The polymers, which could not be identified were classified as NI. Most NI polymers were coloured.

### Processing of sequencing data

Merging of paired‐end reads, quality filtering (max. error of 1 in 100 nucleotides), elimination of singletons, chimera filtering, mapping and clustering of OTU were performed using usearch tools, with OTU clustering being performed by UPARSE (Edgar, 2013). Taxonomic assignments of OTUs were done using blastn against the SILVA_138_SSURef_NR99 reference database with a threshold of 97% similarity between the query and reference sequences. Samples with less than 1000 classified reads were excluded from further analysis. All OTUs classified as chloroplasts were excluded from further analysis.

### Statistical analysis

All statistical analyses were performed using R version 3.5.1 in RStudio version 1.1.456. All plots were done using R package ‘ggplot2’ version 3.3.1 (Wickham, 2016), unless stated otherwise. Venn diagrams were built using the R package ‘VennDiagram’ version 1.6.20 (Chen and Boutros, 2011). OTU diversity and richness indexes were calculated with the R package ‘iNext’ version 2.0.20 (Hsieh *et al*., 2016) using a coverage base rarefaction of 97%, corresponding to the lower coverage calculated for a sample.

To visualize the similarity of the prokaryotic communities of all samples, not including the in situ or the laboratory incubation samples, NMDS was performed with Bray–Curtis dissimilarity as the distance measurement. The environmental factors (temperature, salinity and PO_4_, NO_3_ and NO_2_ concentrations) were fitted to the ordination plots using the ‘envfit’ function of the vegan package in R. A Mantel test was performed to evaluate correlations between the prokaryotic community composition of the biofilms of polymers and the different environmental factors. An additional Mantel test was performed to determine correlations between the relative abundance of the 35 OTUs enriched in the laboratory incubations and environmental factors. Euclidian distance matrices were used for the environmental factors and a Bray–Curtis dissimilarity matrix was used for the community composition. A PERMANOVA was performed to determine whether the prokaryotic community composition was significantly different between sampling regions (e.g. Atlantic Ocean, Pacific Ocean and northern Adriatic Sea), sampling stations or sample type (e.g. PE, PP, PET, nylon and ambient SW). PERMANOVA analysis was also performed just with the 35 OTUs enriched in the laboratory experiments after 1 year of incubation. Samples from polymers remaining unclassified were not considered in this analysis. We also evaluated differences in community composition between different sites, stations and plastic types using homogeneity of dispersions tests on Bray–Curtis dissimilarity matrices (‘betadisper’ from R package ‘vegan’ version 2.5.6). Pair‐wise PERMANOVAs were performed to each of the above‐mentioned factors to determine differences between stations, oceanic basins or sample types. Differences were considered significant if *p* < 0.05.

The fold‐change in abundance of each OTU between sample types for each location was calculated using the R package ‘DESeq2’ (Love *et al*., 2014), which fits negative binomial generalized linear models for each OTU and tests significance using the Wald test.

## Supporting information


**Appendix** **S1.** Supporting Information.

**Table S1.** Information on the sampling site. (.xlsx)

**Table S2.** Information on the samples. (.xlsx)

**Table S5.** Results of the PERMANOVA and pair‐wise PERMANOVAs comparing the prokaryotic community composition between different polymer types, sampling site, sampling station and season in the case of the North Adriatic samples. (.xlsx)

**Table S8.** Relative abundance of families present with more than 1% relative abundance in at least one samples across the three sampling sites. (.xlsx)

**Table S9.** OTUs found in plastics from all sampling sites, but not in SW. (.xlsx)

**Table S10.** OTUs with more than 1% relative abundance in at least one sample. (xlsx)

**Table S11.** OTUs with more than 1% relative abundance in at least one sample of each sampling site. (.xlsx)

**Table S12.** OTUs that were enriched in the laboratory LDPE incubations after one year incubation, and that were still present in incubation B after two years. (.xlsx)
