# Supplementary material for: Putative degraders of low‐density polyethylene‐derived compounds are ubiquitous members of plastic‐associated bacterial communities in the marine environment
Source: Environ Microbiol. 2020 Sep 28;22(11):4779–93. doi: 10.1111/1462-2920.15232 (PMC7702132; doi:10.1111/1462-2920.15232)
Supplement: Supplementary file 1 — Appendix S1. Supporting Information. [file EMI-22-4779-s001.docx]

**Supplementary Information**

**S1**- Results of the taxonomy of the prokaryotic community on plastics and seawater across oceanic regions.

**S2-** Discussion on the comparison of the prokaryotic community composition of plastic biofilms collected at different sites.

**Fig. S1**- Sampling stations in (A) the Pacific Ocean, (B) the North Atlantic and (C) the northern Adriatic Sea.

**Fig. S2-** OTU richness and diversity indexes of samples collected across the three different sites. Each symbol represents one sample.

**Fig. S3** - Venn diagrams displaying the relatively abundant families (>1% relative abundant in at least one sample of that site) shared between the biofilm of plastics collected from the different sampling sites and seawater.

**Fig. S4** - Stacked plot of all families present in at least one sample with a relative abundance >10% in the samples collected in the northern Adriatic.

**Fig. S5**- Stacked plot of all families that were present at least in one sample with relative abundance >10% in the samples collected in the (A) North Atlantic and (B) Pacific.

**Fig. S6** - Venn diagrams displaying OTUs shared between different sites.

**Fig. S7** - Enriched bacterial families between the PE and PP samples collected in the northern Adriatic.

**Fig. S8 –** Photos of the Original plastics A and B collected from the northern Adriatic Sea.

**Fig. S9**- Stacked plot of OTUs that increased in relative abundance in the LDPE-incubations over time.

**Fig. S10** - Sampling nets used to collect plastics in different sites.

**Fig. S11** – Scheme of the LDPE-laboratory incubations.

**Fig. S12** – Stacked plots of the relative abundance of the OTUs with relative abundance >0.7% found in each blank.

**Table S1** - Information on the sampling site. (.xlsx)

**Table S2** - Information on the samples. (.xlsx)

**Table S3** - Results of the ANOVA tests comparing diversity and richness indexes of the prokaryotic communities between the different sites, stations, season (in the case of the North Adriatic samples) and size of the plastics.

**Table S4** - Results of the post-hoc Tukey tests performed on the ANOVAs comparing richness and diversity indexes of the different sampling sites (Table S3).

**Table S5** – Results of the PERMANOVA and pair-wise PERMANOVAs comparing the prokaryotic community composition between different polymer types, sampling site, sampling station and season in the case of the North Adriatic samples. (.xlsx)

**Table S6** – Envfit test results of fitted environmental factors to the ordination scores (NMDS) of samples dependent on their prokaryotic community composition.

**Table S7** - Mantel tests identifying correlations between the community composition of all polymers and the distance between sampling stations and environmental factors.

**Table S8** – Relative abundance of families present with more than 1% relative abundance in at least one samples across the three sampling sites. (.xlsx)

**Table S9** – OTUs found in plastics from all sampling sites, but not in SW. (.xlsx)

**Table S10** – OTUs with more than 1% relative abundance in at least one sample. (xlsx)

**Table S11** – OTUs with more than 1% relative abundance in at least one sample of each sampling site. (.xlsx)

**Table S12** – OTUs that were enriched in the laboratory LDPE incubations after one year incubation, and that were still present in incubation B after two years. (.xlsx)

**S1 – Taxonomy of the prokaryotic community on plastics and seawater across oceanic regions**

There were 11 families that had a relative abundance > 1% in at least one plastic particle in all sampling sites (Fig. S3); these were *Cyanobiaceae, Flavobacteriacea; Hyphomonadaceae, Moraxellaceae, Phormidesmiaceae, Pirellulaceae, Pseudoalteromonadaceae, Rhodobacteraceae, Sphingomonadaceae, Planococcaceae, and Rhizobiaceae*. In contrast to the other families, the latter two were only abundant on plastic and not in any of the seawater samples. Families that were relatively abundant (>1% relative abundance) on almost all plastics were *Rhodobacteraceae*, *Rhizobiaceae*, *Moraxellaceae* and *Hyphomonadaceae* (Table S8n). Together, these 11 families represented a minimum of 45% ± 26% (n= 8) of the total classified 16S rRNA gene sequences of the biofilm communities in the northern Adriatic during the winter and a maximum of 88% ± 5% (n=4) on plastics collected at Station 5 in the North Atlantic.

*Erythrobacter* were relatively abundant in almost all plastic samples across the three oceanic regions (Fig. S4 and Fig. S5). And microbes classified as *Yoonia-Loktanella* were abundant in almost all plastic samples across the Atlantic and Pacific (Fig. S5). Certain taxa like *Sulfitobacter* and *Phormidesmis* were more abundant in the plastics collected in the Atlantic, while others like *Roseovarius* and *Woeseia* were more abundant on plastics collected in the Adriatic (Fig. S4).

**S2- Comparison of the prokaryotic community composition of plastic biofilms collected at different sites**

For the plastic material collected in the different oceanic regions, it is unknown for how long these plastics were floating at the sea-surface. Therefore, the developmental stage of the plastic biofilm cannot be determined. Plastics collected at more coastal sites in the northern Adriatic and in the North Atlantic at Station 5 are more likely to have been in the water for shorter periods of time than plastics collected in the open ocean, like at the Pacific stations (Brandon et al., 2016). This could explain why the community composition differed between stations in the North Atlantic but not in the Pacific samples and why there was a significant correlation between the community composition and distance to land in the North Atlantic (Table S7). The stations in the North Atlantic ranged from more coastal to open ocean, while the Pacific stations were all in the open ocean (Fig. S1). Coastal stations are also more likely to harbour a mixture of plastics with some floating at the surface already for a long time, while other plastics might be in the water for only a short period. A higher variability in the time the plastics collected in the northern Adriatic spent in the water could also explain the higher variability in the community composition and OTU diversity between different seasons (Fig. 1, Fig. S2). However, the lack of correlations between environmental factors and the prokaryotic community composition at the stations in the Pacific and North Atlantic could also be the result of plastics present in the water at a specific site for a variable period of time. Thus, biofilms could have been collected at different stages of development and differently influenced by environmental factors. Furthermore, plastic exposure to UV radiation (Pinto et al., 2019), presence of eukaryotes (von Ammon et al., 2018) and plastic-related characteristics, such as the presence of additives and other leachable compounds (Romera-Castillo et al., 2018; Tetu et al., 2019), sorption of compounds from the water (Rochman et al., 2013) and physico-chemical properties (Gross et al., 2016) are also likely to shape the microbial community of plastics biofilms.


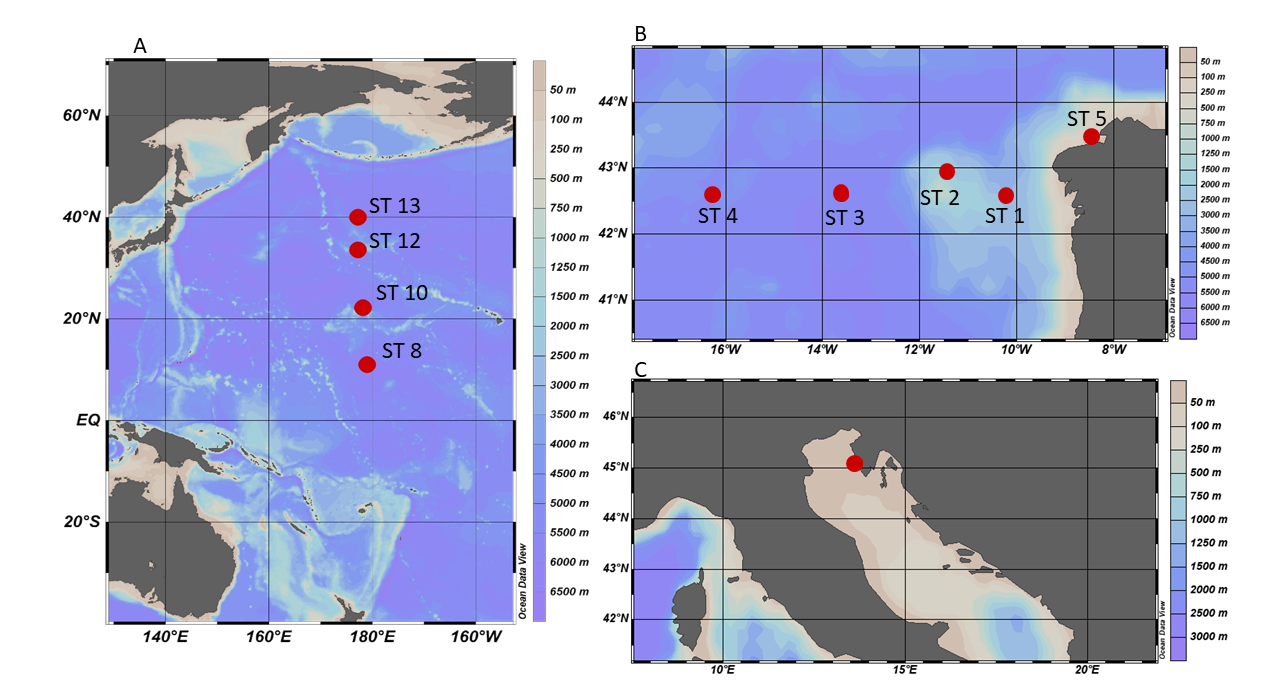


Fig. S1- Sampling stations in (A) the Pacific Ocean, (B) the North Atlantic and (C) the northern Adriatic Sea.


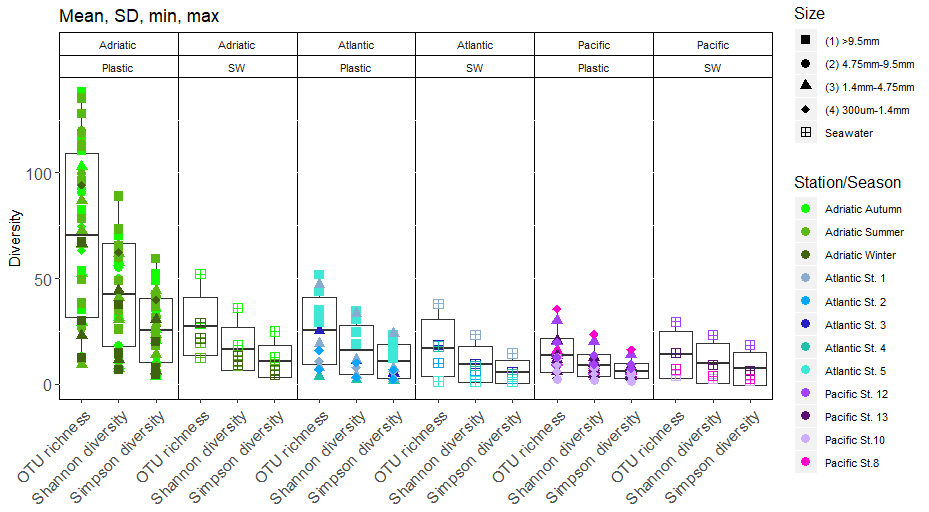


Size

Fig. S2- OTU richness and diversity indexes of samples collected across the three different sites. Each symbol represents one sample. The boxplots represent the mean ± SD and the whiskers represent the minimum and maximum values.


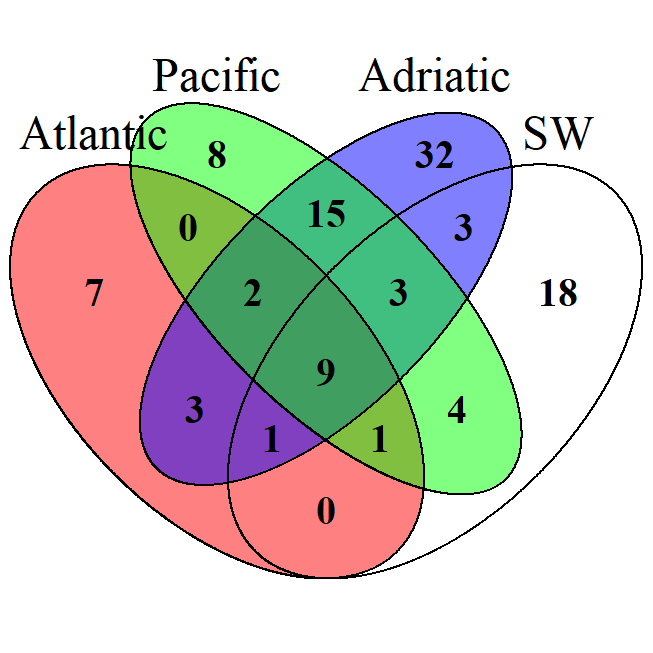


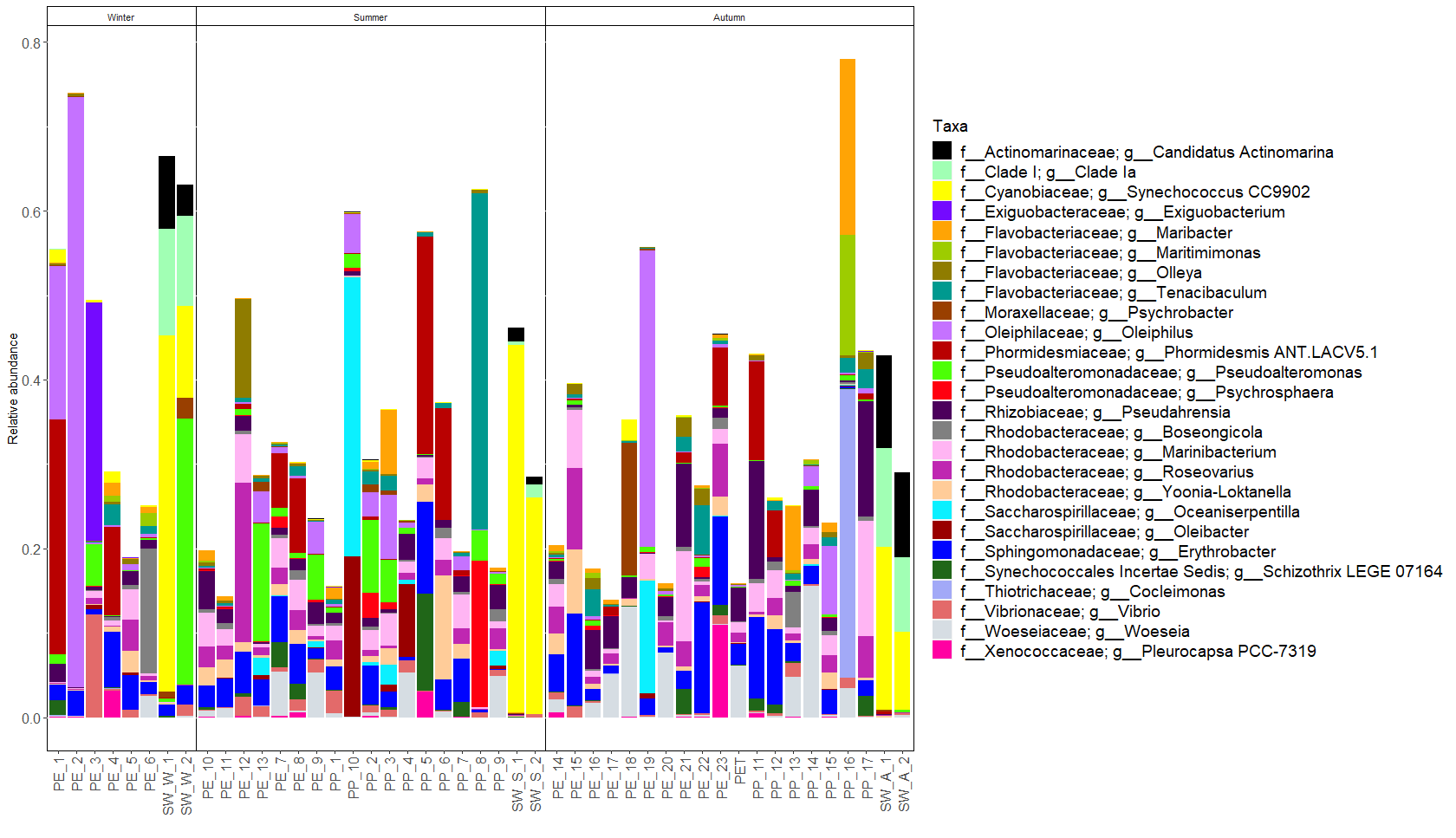
Fig. S3 - Venn diagrams displaying the relatively abundant families (>1% relative abundant in at least one sample of that site) shared between the biofilm of plastics collected from the different sampling sites and seawater (SW). The SW category includes all seawater samples from the considered sites combined.

Fig. S4 - Stacked plot of genera present in at least one sample with a relative abundance >10% in the samples collected in the northern Adriatic.


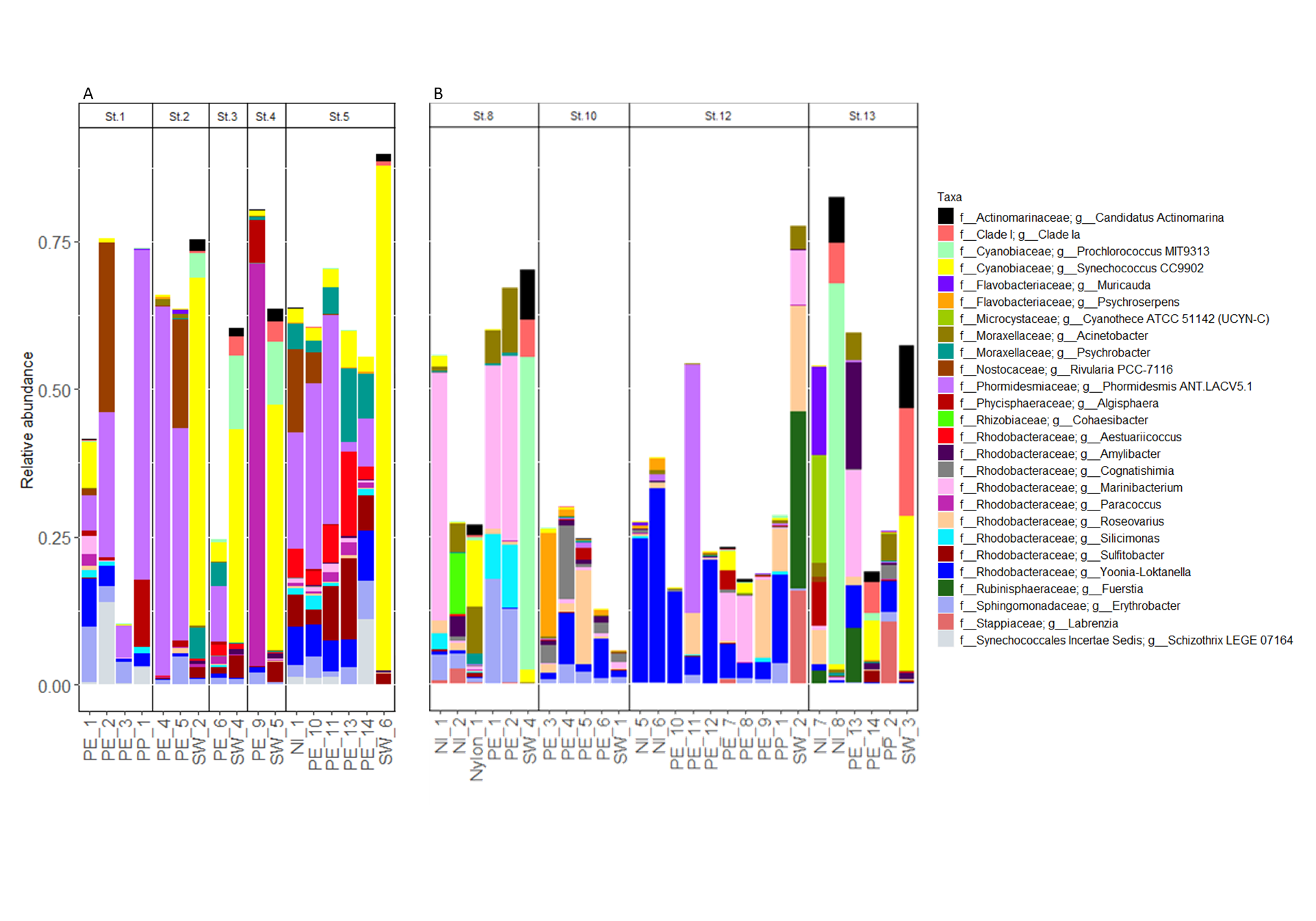


Fig. S5- Stacked plot of the genera present in at least one sample with relative abundance >10% in the samples collected in the (A) North Atlantic and (B) Pacific.


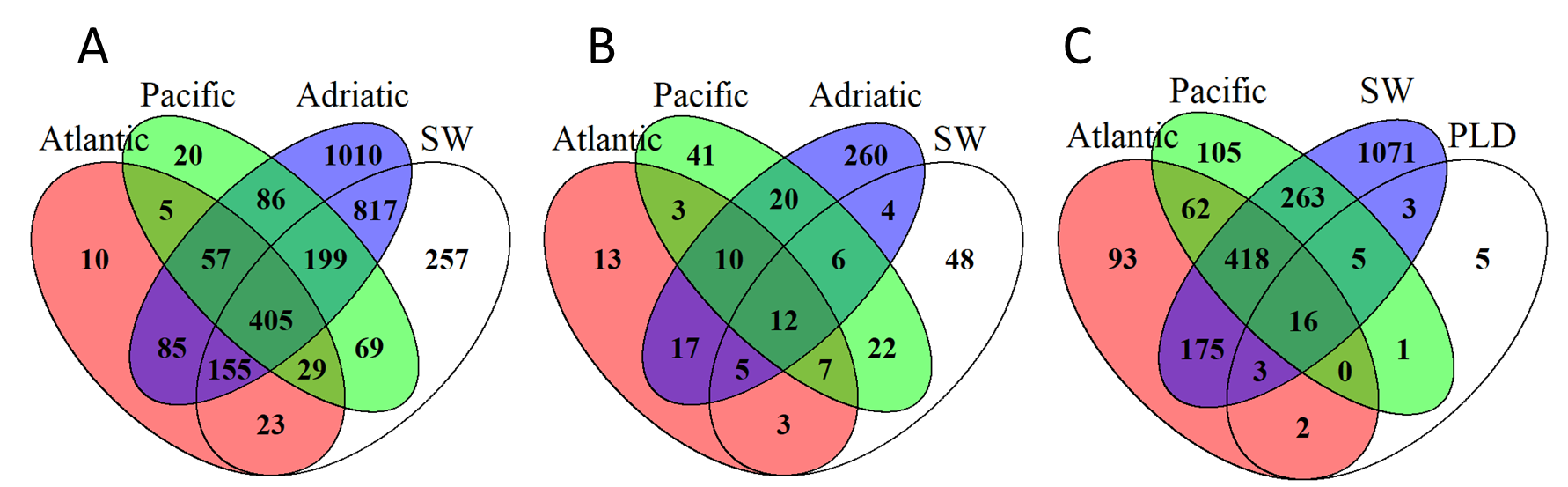


Fig. S6 - Venn diagrams displaying: (A) the OTUs shared between the biofilm of plastics collected at the different sampling sites and seawater; (B) the relatively abundant OTUs (>1% relative abundance in at least one sample at the specific site) shared between the biofilm of plastics collected at the different sampling sites and seawater, and (C) the potential LDPE-degrading OTUs (PLD) enriched in the LDPE laboratory incubations and the OTUs from the biofilms of plastics collected in the Pacific and North Atlantic. The SW category included all seawater samples from the respective sites combined.


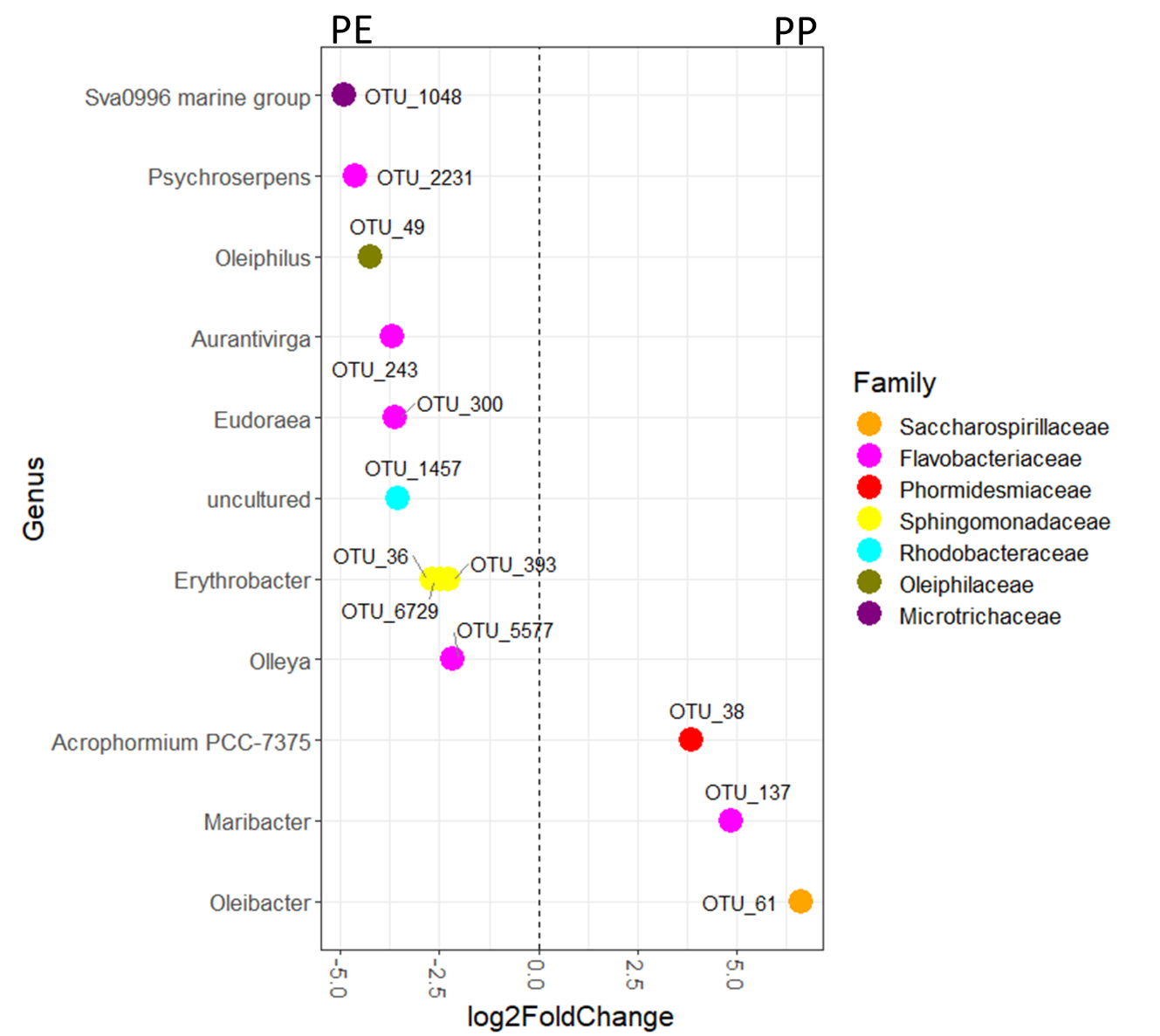


Fig. S7 - Enriched bacterial families between the PE and PP samples collected in the northern Adriatic. OTUs with fold change > 0 were enriched in PP, and OTUs with fold change < 0 were enriched in PE. Each symbol represents one OTU. Only statistically and significantly enriched taxa were included (adjusted p-value < 0.05).


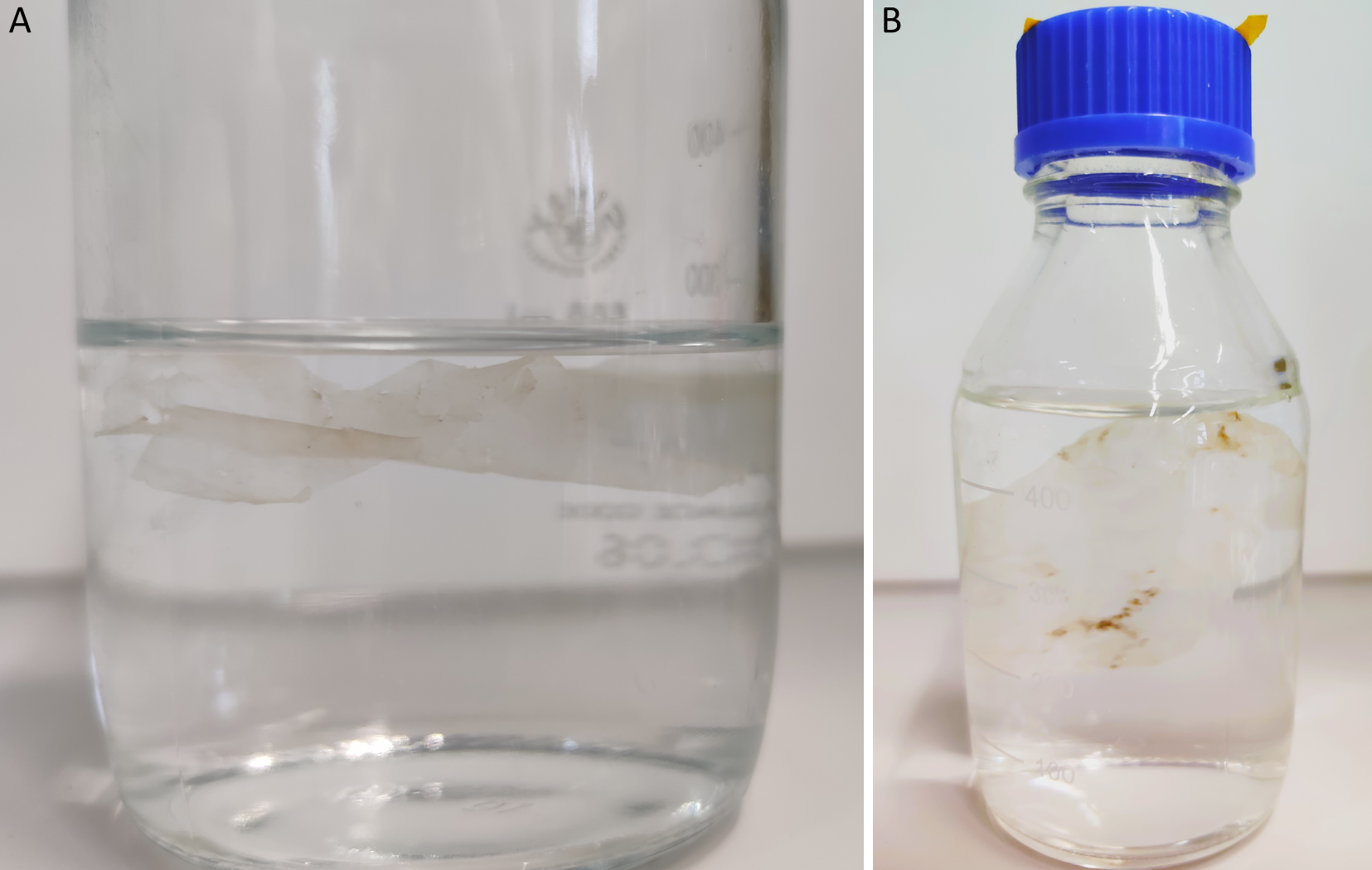


Fig. S8 – Original plastics A (A) and B (B) collected from the northern Adriatic Sea.


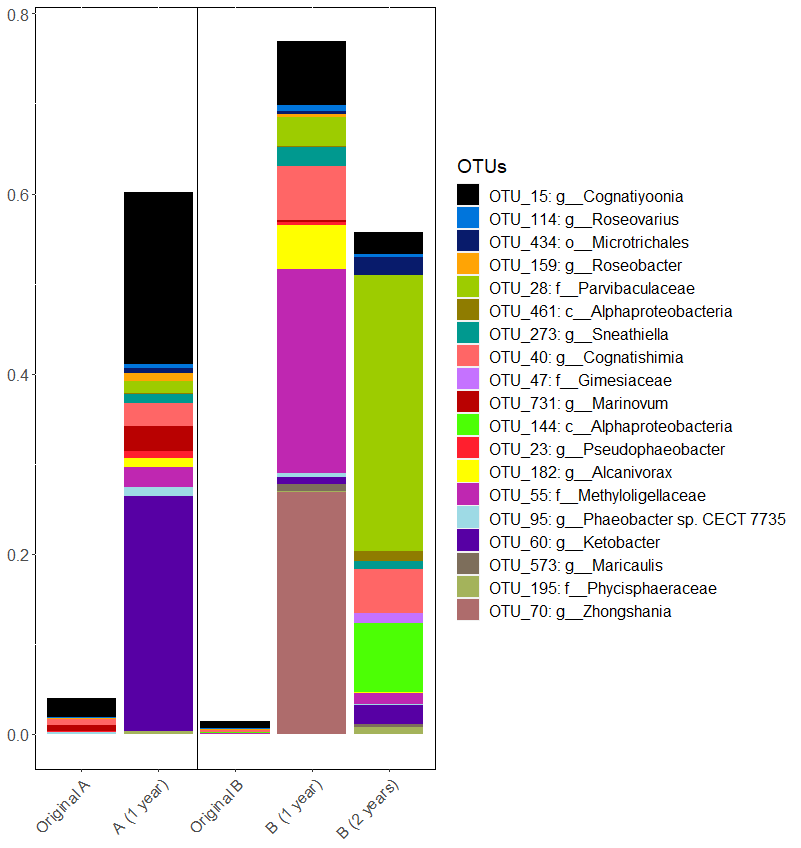


Fig. S9- Stacked plot of the potential LDPE-degrading OTUs. Potential LDPE-degrading OTUs are tentatively defined as OTUs that increased in relative abundance in the biofilm of both LDPE incubations A and B after one year and present in incubation B after two years. The y-axis represents the relative abundance, and the x-axis represents the different stages of incubations A and B. Original A and B samples refer to the original prokaryotic community of the biofilm of the original plastics collected in the North Adriatic. Only OTUs that occurred with relative abundance >0.5% in at least on of the samples are represented.


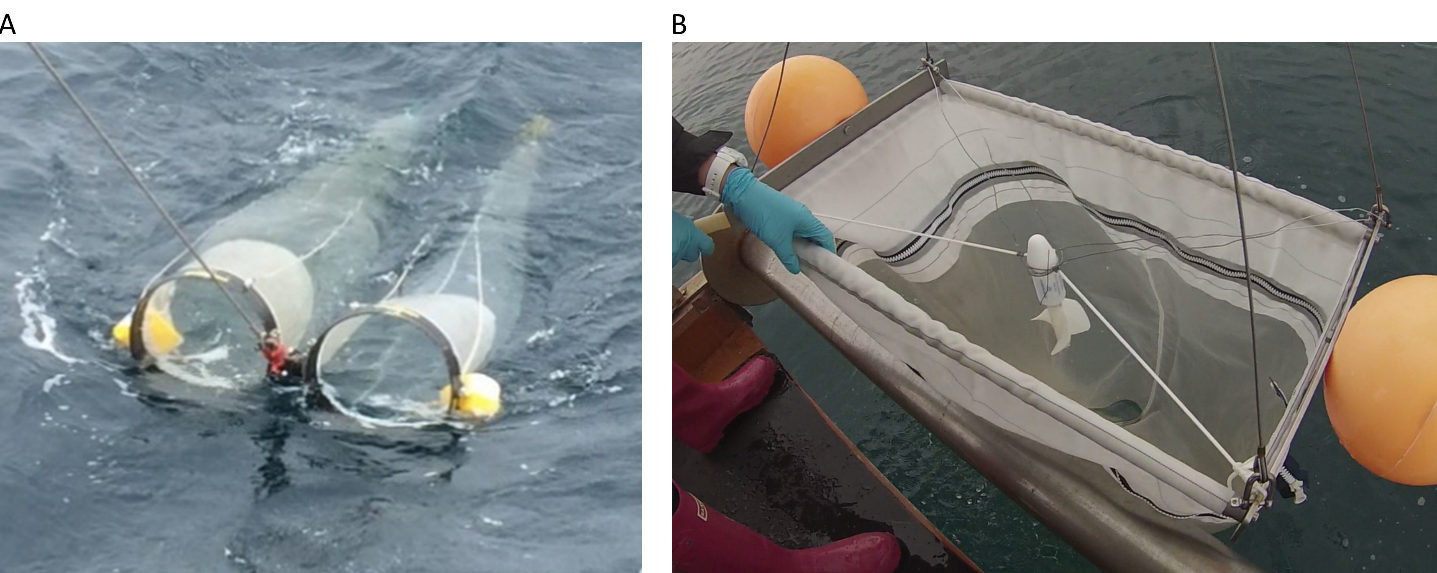


Fig. S10 - Sampling nets used to collect plastics in different sites. (A) Bongo net used to collect plastic samples during the SO248 expedition in the Pacific. (B) Manta net for collecting microplastic used for sampling in the northern Adriatic and during the Radprof expedition in the North Atlantic.


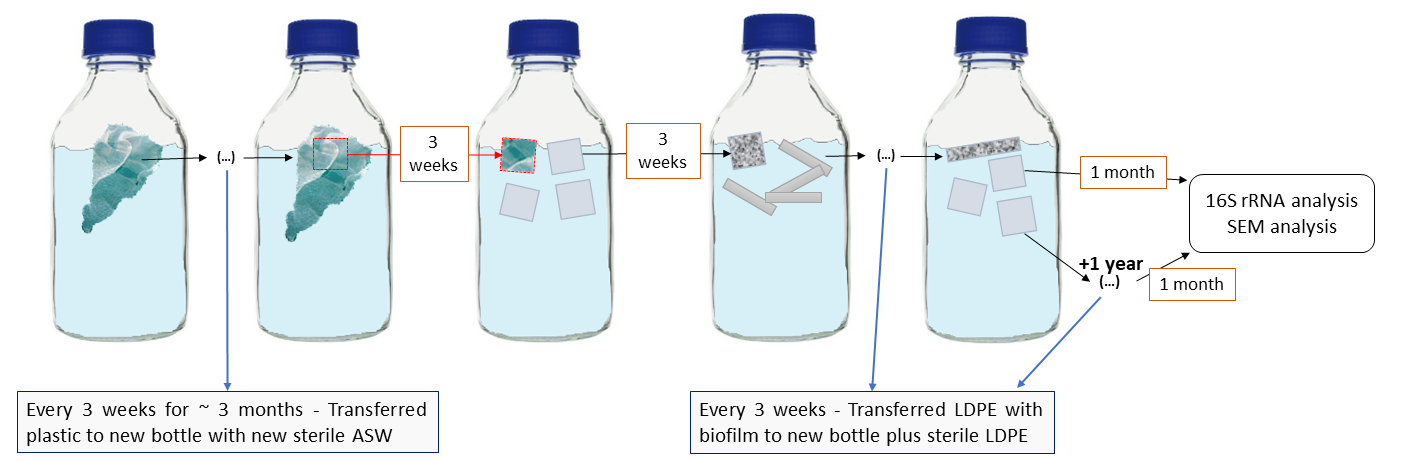


Fig. S11 – Scheme of the LDPE-laboratory incubations. The shapes with a black and white pattern represent LDPE pieces with biofilm.


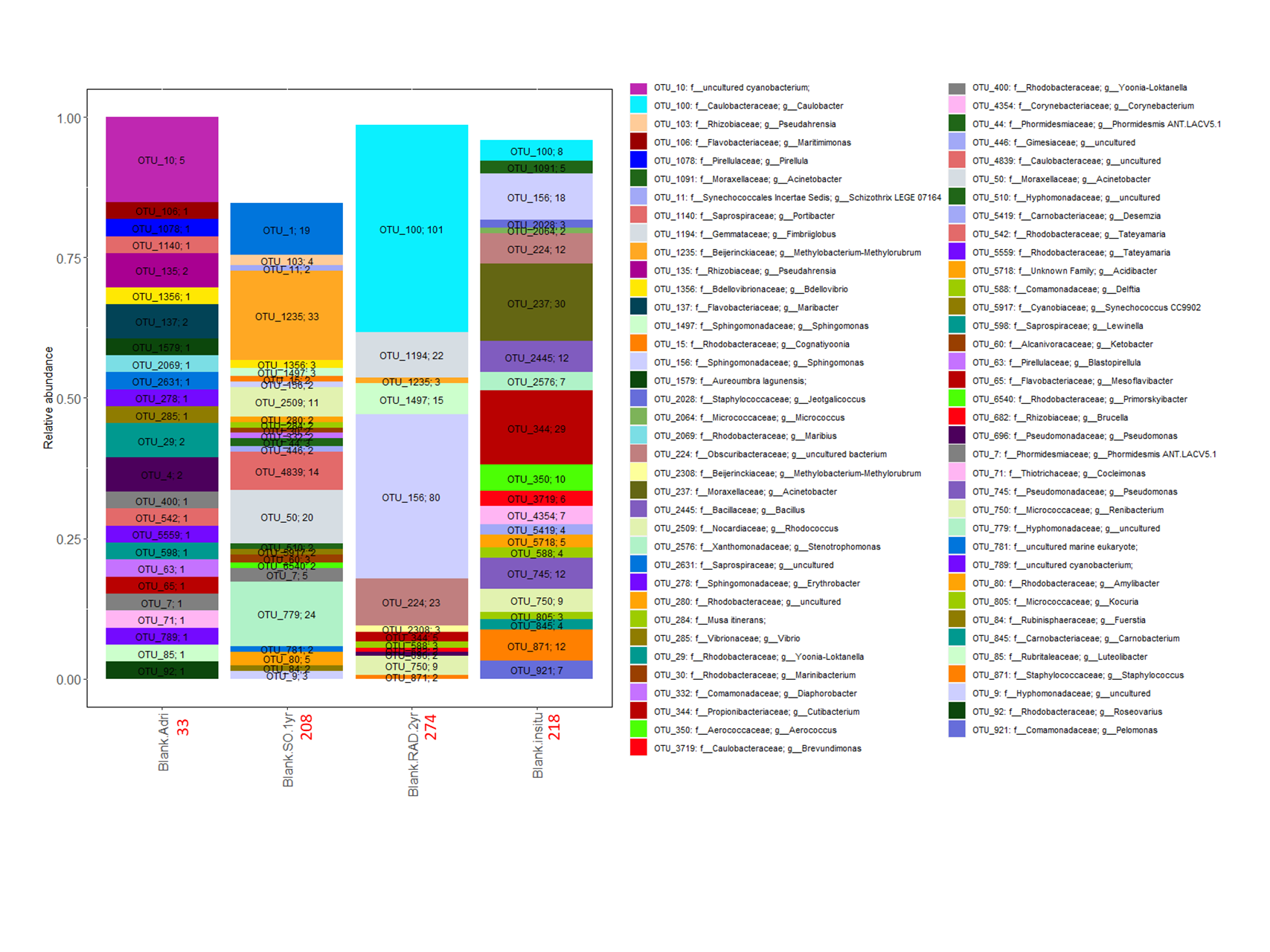


Fig. S12 – Relative abundance of the OTUs with relative abundance >0.7% found in each blank. The text in the in the plot refers to the OTU number and the number of respective classified reads. The red numbers represent the total number of analysed reads of each sample.

Table S3- Results of the ANOVA tests comparing diversity and richness indexes of the prokaryotic communities between the different sites, stations, season (in the case of the northern Adriatic samples) and size of the plastics. * indicates p < 0.05.

|  | **OTU Richness** | | **Shannon index** | | **Simpson index** | |
| --- | --- | --- | --- | --- | --- | --- |
|  | **F value** | **p-value** | **F value** | **p-value** | **F value** | **p-value** |
| **Site** | 29.888 | **2.04E-09*** | 26.258 | **1.19E-08*** | 22.378 | **9.00E-08*** |
| **Season/Station** | 0.787 | 0.629 | 0.753 | 0.659 | 0.72 | 0.689 |
| **Size** | 1.11 | 0.353 | 1.476 | 0.232 | 2.153 | 0.105 |
| **Site:Size** | 0.171 | 0.916 | 0.273 | 0.844 | 0.37 | 0.775 |
| **Season/Station:Size** | 1.621 | 0.16 | 1.339 | 0.256 | 1.621 | 0.16 |

Table S4 – Results of the post-hoc Tukey tests performed on the ANOVAs comparing richness and diversity indexes of the different sampling sites (Table S3). * indicates p < 0.05.

|  | **OTU Richness** | | **Shannon index** | | **Simpson** | |
| --- | --- | --- | --- | --- | --- | --- |
|  | **diff** | **p adj** | **diff** | **p adj** | **diff** | **p adj** |
| **Atlantic-Adriatic** | -45.221 | **5.22E-05*** | -26.149 | **1.76 E-04*** | -14.467 | **7.40E-04*** |
| **Pacific-Adriatic** | -56.812 | **0.000*** | -33.457 | **0.000*** | -19.140 | **2E-07*** |
| **Pacific-Atlantic** | -11.590 | 0.512 | -7.3085 | 0.507546 | -4.672 | 0.481 |

Table S6- Envfit test results of fitted environmental factors to the ordination scores (NMDS) of samples dependent on their prokaryotic community composition. NMDS analyses were conducted for all samples (Fig. 1), all polymer samples excluding seawater samples, and for only the polymers we could identify. * indicates p < 0.05.

|  |  | **Pacific** | | **Atlantic** | | **Adriatic** | | **Atlantic + Adriatic** | | **All** | |
| --- | --- | --- | --- | --- | --- | --- | --- | --- | --- | --- | --- |
|  |  | **R2** | **p-value** | **R2** | **p-value** | **R2** | **p-value** | **R2** | **p-value** | **R2** | **p-value** |
| **All samples** | **Temperature** | 0.431 | **0.002*** | 0.276 | 0.075 | 0.113 | 0.072 | 0.059 | 0.157 | 0.063 | **0.049*** |
|  | **Salinity** | 0.010 | 0.864 | 0.218 | 0.154 | 0.045 | 0.33 | 0.051 | 0.192 | 0.120 | **0.002*** |
|  | **NO2** | **-** | - | 0.349 | **0.038*** | 0.096 | 0.117 | **0.431** | **0.001*** | **-** | - |
|  | **NO3** | **-** | - | 0.309 | 0.064 | 0.076 | 0.19 | **0.489** | **0.001*** | **-** | - |
|  | **PO4** | - | - | 0.278 | 0.099 | 0.021 | 0.619 | **0.316** | **0.001*** | - | - |
| **Just polymers** | **Temperature** | 0.111 | 0.319 | 0.128 | 0.519 | 0.092 | 0.179 | 0.169 | **0.013*** | 0.034 | 0.284 |
|  | **Salinity** | 0.231 | 0.062 | 0.101 | 0.585 | 0.092 | 0.179 | 0.107 | 0.056 | 0.066 | 0.076 |
|  | **NO2** | **-** | - | 0.436 | 0.055 | 0.092 | 0.179 | 0.473 | **0.001*** | **-** | - |
|  | **NO3** | **-** | - | 0.504 | **0.032*** | 0.092 | 0.179 | 0.471 | **0.001*** | **-** | - |
|  | **PO4** | - | - | 0.444 | 0.053 | 0.092 | 0.179 | 0.015 | **0.001*** | - | - |
| **Polymers excluding NI** | **Temperature** | 0.066 | 0.613 | 0.16 | 0.47 | 0.092 | 0.179 | 0.115 | **0.042*** | 0.073 | 0.06 |
|  | **Salinity** | 0.080 | 0.552 | 0.081 | 0.68 | 0.092 | 0.179 | 0.065 | 0.178 | 0.063 | 0.108 |
|  | **NO2** | **-** | - | 0.610 | **0.012*** | 0.092 | 0.179 | 0.441 | **0.001*** | **-** | - |
|  | **NO3** | **-** | - | 0.615 | **0.012*** | 0.092 | 0.179 | 0.461 | **0.001*** | **-** | - |
|  | **PO4** |  | - | 0.247 | 0.294 | 0.092 | 0.179 | 0.221 | **0.003*** | - | - |

Table S7- Mantel tests identifying correlations between the community composition of all polymers and the distance between sampling stations and environmental factors. SW samples were excluded from this analysis. The analysis was repeated considering only the relative abundance of the OTUs enriched in the laboratory LDPE incubations (potential LDPE degraders). Pacific samples were excluded from all the inorganic nutrients analyses (indicated with ^†^). * indicates p < 0.05.

|  |  | **Pacific** | | **Atlantic** | | **Adriatic** | | **All** | |
| --- | --- | --- | --- | --- | --- | --- | --- | --- | --- |
|  |  | **R2** | **p-value** | **R2** | **p-value** | **R2** | **p-value** | **R2** | **p-value** |
| **All OTUs**  **(n=3352)** | **Temperature** | -0.142 | 0.75 | 0.103 | 0.391 | -0.5 | 0.833 | -0.344 | 0.99 |
|  | **Salinity** | 0.942 | 0.083 | -0.284 | 0.633 | -0.5 | 0.833 | -0.197 | 0.847 |
|  | **NO2** | - | - | -0.454 | 0.866 | 0.5 | 0.5 | -0.188^†^ | 0.691^†^ |
|  | **NO3** | - | - | -0.329 | 0.725 | 1 | 0.167 | -0.046^†^ | 0.479^†^ |
|  | **PO4** | **-** | - | 0.309 | 0.283 | -0.5 | 0.833 | -0.166^†^ | 0.658^†^ |
|  | **Distance** | -0.371 | 0.916 | 0.697 | **0.025*** | - | - | -0.003 | 0.435 |
|  | **Distance to land** | - | - | 0.951 | **0.008*** | - | - | - | - |
| **Potential LDPE degraders (n=35)** | **Temperature** | -0.085 | 0.682 | -0.393 | 0.925 | -0.5 | 0.833 | -0.284 | 0.976 |
|  | **Salinity** | -0.142 | 0.5 | -0.49 | 0.991 | 1 | 0.166 | -0.088 | 0.636 |
|  | **NO2** | - | - | -0.103 | 0.6 | -1 | 1 | -0.255^†^ | 0.845^†^ |
|  | **NO3** | - | - | 0.122 | 0.375 | -0.5 | 0.833 | -0.153^†^ | 0.632^†^ |
|  | **PO4** | **-** | - | 0.927 | **0.008*** | 1 | 0.166 | 0.039^†^ | 0.417^†^ |
|  | **Distance** | 0.257 | 0.291 | 0.684 | 0.066 | - | **-** | 0.068 | 0.276 |
|  | **Distance to land** | - | - | 0.952 | **0.008*** | - | **-** | - | - |

**References**

Brandon, J., Goldstein, M., & Ohman, M. D. (2016). Long-term aging and degradation of microplastic particles: Comparing in situ oceanic and experimental weathering patterns. *Mar Pollut Bull* **110**: 299–308.

Gross, M., Zhao, X., Mascarenhas, V., & Wen, Z. (2016). Effects of the surface physico-chemical properties and the surface textures on the initial colonization and the attached growth in algal biofilm. *Biotechnol Biofuels* **9**: 38.

Pinto, M., Langer, T. M., Hüffer, T., Hofmann, T., & Herndl, G. J. (2019). The composition of bacterial communities associated with plastic biofilms differs between different polymers and stages of biofilm succession. *PloS One* **14**: e0217165.

Rochman, C. M., Hoh, E., Hentschel, B. T., & Kaye, S. (2013). Long-term field measurement of sorption of organic contaminants to five types of plastic pellets: implications for plastic marine debris. *Environ Sci Technol* **47**: 1646–1654.

Romera-Castillo, C., Pinto, M., Langer, T. M., Álvarez-Salgado, X. A., & Herndl, G. J. (2018). Dissolved organic carbon leaching from plastics stimulates microbial activity in the ocean. *Nat Commun* **9**: 1-7.

Tetu, S. G., Sarker, I., Schrameyer, V., Pickford, R., Elbourne, L. D. H., Moore, L. R., & Paulsen, I. T. (2019). Plastic leachates impair growth and oxygen production in Prochlorococcus, the ocean’s most abundant photosynthetic bacteria. *Commun Biol* **2**: 1-9.

von Ammon, U., Wood, S. A., Laroche, O., Zaiko, A., Tait, L., Lavery, S., *et al*. (2018). The impact of artificial surfaces on marine bacterial and eukaryotic biofouling assemblages: a high-throughput sequencing analysis. *Mar Environ Res* **133:** 57–66.
